# Synthesis of Triazole-Linked SAM-Adenosine Conjugates: Functionalization of Adenosine at N-1 or N-6 Position without Protecting Groups

**DOI:** 10.3390/molecules25143241

**Published:** 2020-07-16

**Authors:** Colette Atdjian, Dylan Coelho, Laura Iannazzo, Mélanie Ethève-Quelquejeu, Emmanuelle Braud

**Affiliations:** Laboratoire de Chimie et Biochimie Pharmacologiques et Toxicologiques, Université de Paris, CNRS UMR 8601, F-75006 Paris, France; colette.atdjian@wanadoo.fr (C.A.); dylan.coelho@etu.parisdescartes.fr (D.C.)

**Keywords:** RNA m^6^A methyltransferase, RNA m^1^A methyltransferase, bisubstrate analogues, *S*-adenosyl-l-methionine, 1-*N*-alkylated adenosine, 1,2,3-triazole, click chemistry, CuAAC

## Abstract

More than 150 RNA chemical modifications have been identified to date. Among them, methylation of adenosine at the N-6 position (m^6^A) is crucial for RNA metabolism, stability and other important biological events. In particular, this is the most abundant mark found in mRNA in mammalian cells. The presence of a methyl group at the N-1 position of adenosine (m^1^A) is mostly found in ncRNA and mRNA and is mainly responsible for stability and translation fidelity. These modifications are installed by m^6^A and m^1^A RNA methyltransferases (RNA MTases), respectively. In human, deregulation of m^6^A RNA MTases activity is associated with many diseases including cancer. To date, the molecular mechanism involved in the methyl transfer, in particular substrate recognition, remains unclear. We report the synthesis of new SAM-adenosine conjugates containing a triazole linker branched at the N-1 or N-6 position of adenosine. Our methodology does not require protecting groups for the functionalization of adenosine at these two positions. The molecules described here were designed as potential bisubstrate analogues for m^6^A and m^1^A RNA MTases that could be further employed for structural studies. This is the first report of compounds mimicking the transition state of the methylation reaction catalyzed by m^1^A RNA MTases.

## 1. Introduction

Among the numerous post-transcriptional modifications of RNA identified to date, methylation is currently one of the most studied [1]. This modification can occur at the terminal cap of RNAs or at internal position, and the methyl group is found on nucleic acid bases or at the 2′ position of the ribose units. The adenine base can be methylated at the C-2 and C-8 atoms as well as at the nitrogen atoms N-1 and N-6 [2,3]. *N*^6^-Methyladenosine (m^6^A) is an abundant reversible modification found in all types of RNA, involved in the regulation of RNA metabolism, protein expression or RNA-protein recognition [4,5,6,7]. Abnormal methylation process is associated with the development of diseases such as cancers, obesity, infertility [8,9,10]. Though less studied, the modification at the N-1 site (m^1^A) is also reversible and linked to the structural stability and the functions of RNAs [11,12].

Since m^1^A can rearrange to m^6^A under alkaline conditions by Dimroth rearrangement [13], its presence in mRNA has been detected only recently in mammalian mRNAs [14,15,16]. The functional consequence of this modification is poorly understood but could impact the regulation and the function of m^1^A for some positions in rRNA and tRNA in the human transcriptome. However, the unique chemical properties of m^1^A, with both a positive charge and a methyl group, potentially allow for a strong effect in terms of RNA structure or protein-RNA interaction [14].

**Scheme 1:**
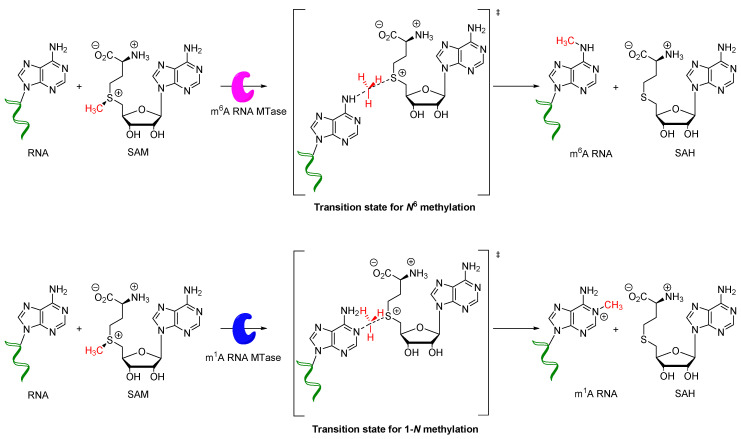
RNA MTases-catalyzed methylation of adenosine at the N-6 and N-1 positions.

For these two modifications (m^1^A and m^6^A), the methyl group is introduced enzymatically by RNA methyltransferases (RNA MTases) that catalyze the transfer of the methyl from the cofactor *S*-adenosyl-l-methionine (SAM) to the nucleotides (Scheme 1). Most m^6^A marks in mammals mRNA are written by a dedicated methyltransferase complex involving the heterodimer METTL3/METTL14, targeting the consensus sequence RRACH [17,18,19]. However, little is known about the recognition of the RNA targets by this complex [20]. METTL16 has a distinct set of targets for m^6^A modification, including the 3′ UTR of MAT2A mRNA and the U6 snRNA, a longer conserved sequence of UACAGAGAA [21,22,23]. Among human m^6^A RNA MTases, only METTL16 has been crystallized with RNA substrate [24]. For m^1^A modification of mRNA it is not clear whether or not specific methylation machinery could also exist. It has been recently hypothesized that the *t*RNA methyltransferase complex TRMT6/61A could catalyze the methyl transfer on the mRNA [16]. In conclusion, few RNA-bound MTases structures are currently available. This lack of complex structures is due to the difficulties in crystallizing RNA/protein complexes. As a consequence, RNA recognition patterns and methylation reaction mechanisms remain poorly understood.

In this context, we recently described the synthesis of SAM-adenosine conjugates as first transition state analogues for m^6^A RNA MTases and their use as tools for structural study [25,26]. We showed that a SAM-adenosine conjugate containing a three-carbon linker tethering the analogue of SAM to the N-6 atom of the adenosine binds the bacterial RNA MTase RlmJ with a conformation close to the real transition state. The structure of this bisubstrate analogue favors the correct positioning of the RNA moiety mimicked by an adenosine and the methionine part of the cofactor into the catalytic site of the MTase. However, a deviation was observed for the positioning of the adenosine in the cofactor part, which is rotated 120° out of the canonical binding pocket for SAH. This deviation indicates that our bisubstrate analogues are not optimal. In this study, we pursue the development of SAM-adenosine conjugates for m^6^A RNA MTases and extend our work to the synthesis of the first potential bisubstrate analogues for m^1^A RNA MTases by covalently linking an analogue of SAM to the N-6 or N-1 atom of the adenosine substrate, respectively.

*N*^6^-alkylation of adenosine derivatives is mainly achieved through aromatic nucleophilic substitution (S_N_Ar) of diverse electrophilic adenosine derivatives [27,28,29,30,31,32,33,34,35,36,37,38,39] or by Dimroth rearrangement of 1-*N*-alkylated adenosines [40,41,42,43,44,45,46,47,48,49,50,51,52]. In the context of S_N_Ar, synthetic strategies involving non classic leaving groups were developed using peptide-coupling agents for activation of the amide group of inosine derivatives [53,54,55,56]. Another study reports the reduction of *N*^6^-acyl-adenosine derivatives with LiAlH_4_ [57]. Selective *N*^6^-alkylation of adenosine derivatives can also be achieved under phase transfer catalysis conditions as described by Arimoto et al. [58]. Two groups used Mitsunobu reaction applied to *N*^6^-acetyl-2′,3′,5′-tri-*O*-acetyladenosine [59,60] or *N*^6^-Boc protected adenosine [61] to regioselectively synthesize *N*^6^-alkylated products. Finally, functionalization of 6-chloroadenosine derivatives can be achieved by palladium-catalyzed Buchwald–Hartwig coupling [62,63].

To date, the alkylation reaction at the N-1 position has been principally developed through the N-1 nucleophilic attack of alkyl halides [40,41,42,43,44,45,46,47,48,49,50,51,52]. This approach allows for the introduction of methyl and alkyl groups as well as benzyl, or allyl substituents. Propargyl group has been also installed on *N*^6^-acetyl-2′,3′,5′-tri-*O*-acetyladenosine but in a quite low yield of 14% [51]. In 2005, Terrazas et al. synthesized 1-*N*-alkylated adenosines by reacting electrophilic inosine with primary amines [64].

**Figure 1:**
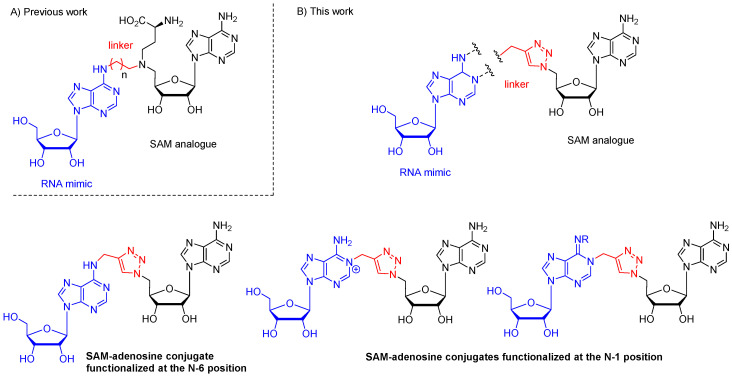
(**A**) Our previous work: structure of SAM-adenosine conjugates with an alkyl linker [25,26]; (**B**) structure of SAM-adenosine conjugates with a 1,2,3-triazole linker synthesized in this study.

In this context, we sought to introduce new chemical modifications at the N-6 and N-1 positions of adenosine to synthesize new SAM-adenosine conjugates. Click chemistry, especially copper(I)-catalyzed alkyne-azide cycloaddition (CuAAC), is an efficient strategy to rapidly synthesize complex structures. On these bases, we designed new bisubstrate analogues bearing a 1,2,3-triazole ring instead of the alkyl linker to increase rigidity between the mimic of the substrate and the SAM analogue while maintaining an appropriate length between the two entities (Figure 1). The synthetic strategy used to obtain these molecules relies on the efficient introduction of the propargyl group at the N-6 and N-1 positions of adenosine derivatives.

## 2. Results and discussion

### 2.1. Synthesis of SAM-Adenosine Conjugates Using Protecting Groups

**Scheme 2:**
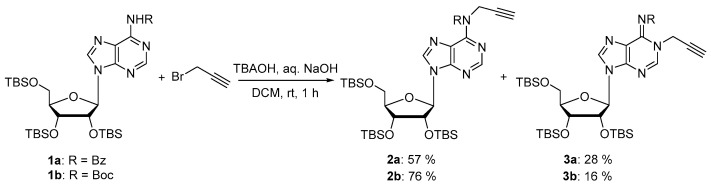
Synthesis of *N*^6^ and 1-*N*-propargylated adenosines.

**Figure 2:**
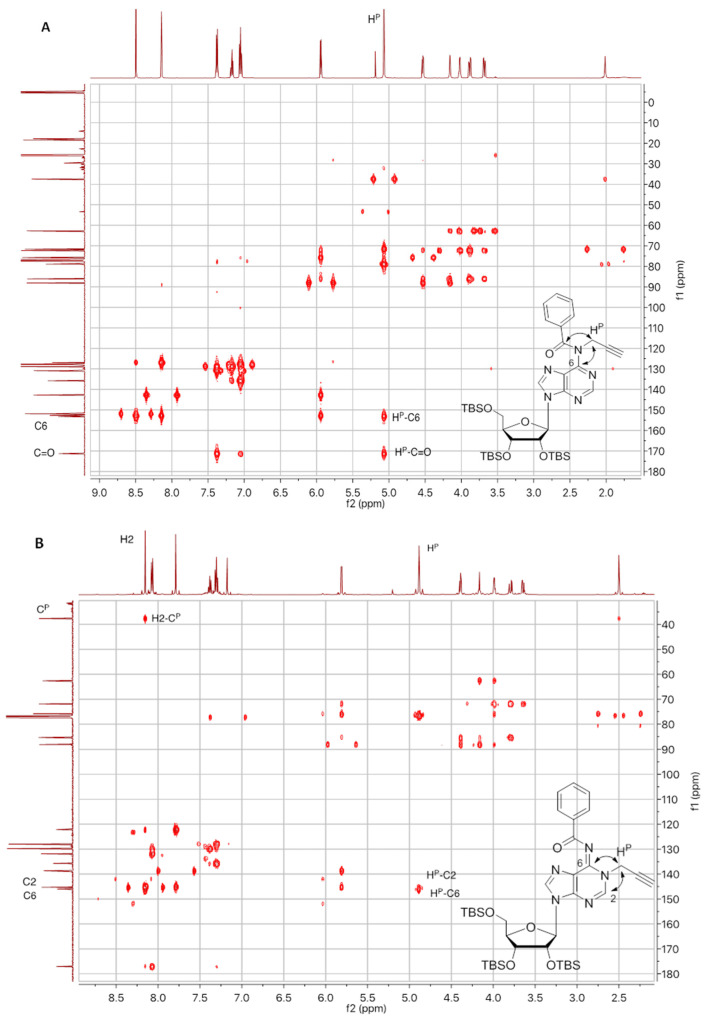
2D HMBC spectra (500 MHz, CDCl_3_): (**A**) *N*^6^-propargyladenosine derivative **2a**. (**B**) 1-propargyladenosine derivative **3a**.

First, we took advantage of the work of Sekine et al. who used tetrabutylammonium bromide (TBABr) as the phase transfer catalyst to produce a mixture of *N*^6^ and 1-*N*-alkylated adenosines from *N*^6^-benzoylated adenosine [58]. A short study was carried out to investigate the alkylation of *N*^6^-benzoyl-2′,3′,5′-tris-*O*-(*tert*-butyldimethylsilyl)adenosine **1a** [65] with propargyl bromide (Scheme 2). Using tetrabutylammonium hydroxide (TBAOH) instead of TBABr led to the formation of adenosines **2a** and **3a** in 57 and 28% yield with total conversion of the starting material (Scheme 2). Analysis of 1D and 2D NMR spectra confirmed the site of alkylation for each regioisomer (Figure 2) [51,58]. The signals for 2-H and 8-H appear at 8.49 and 8.14 ppm for *N*^6^-alkylated compound **2a** while they are upfield at 8.24 and 7.88 ppm in **3a** as expected. Moreover, two correlations are observed in the HMBC spectrum of **2a** between protons of the methylene group of the propargyl (H^p^) and C6 and C=O of benzoyl group in the N-6 regioisomer (Figure 2A). For compound **3a**, HMBC experiments show a correlation between 2-H and the carbon of the methylene group (C^p^) as well as two correlations between the H^p^ protons and C2 and C6 (Figure 2B). The same reaction conditions applied to compound **1b** [25] containing a *tert*-butoxycarbonyl protecting group at the N-6 position of adenosine afforded **2b** and **3b** in 76 and 16% yield respectively, the presence of the carbamate function favoring the *N*^6^ alkylation.

**Scheme 3:**
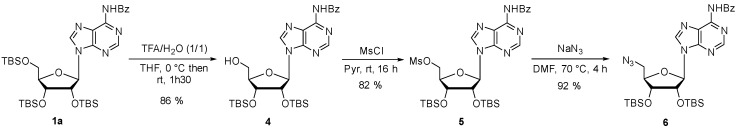
Synthesis of azide **6**.

Azide **6** was synthesized in three steps [25] (Scheme 3). Briefly, removal of TBS group at the 5′ position of **1a** led to the alcohol **4** that was mesylated to afford derivative **5** in 82% yield. Finally, treatment of **5** with sodium azide provided azido adenosine **6** in 92% yield.

**Scheme 4:**
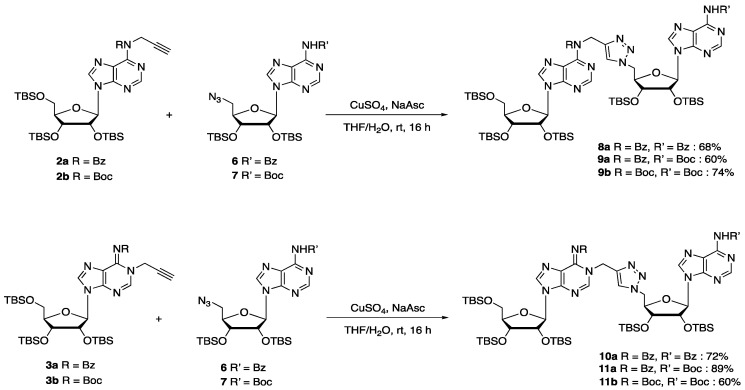
Synthesis of triazoles **8**–**11**.

CuAAC reactions between adenosines **2a**-**b** and **3a**-**b** and azides **6** or **7** [25] were conducted under classic conditions in the presence of sodium ascorbate and copper sulfate in THF/H_2_O to afford triazoles **8**–**11** in 60–89% yield (Scheme 4).

**Scheme 5:**
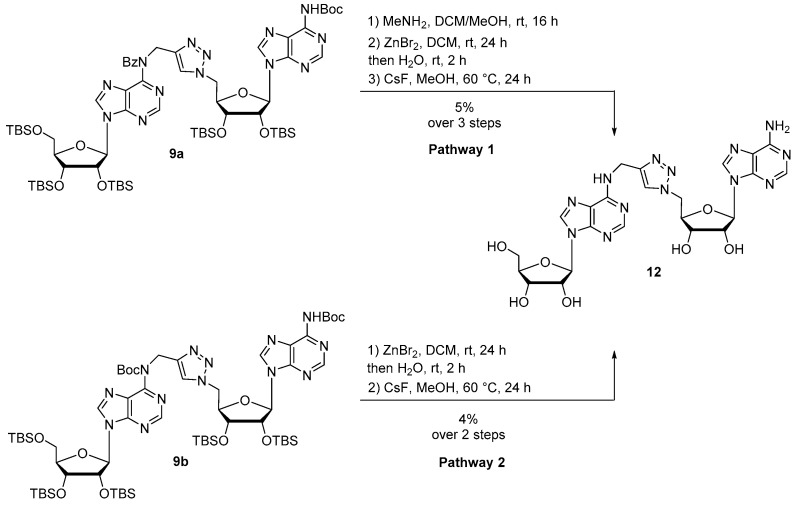
Synthesis of SAM-adenosine conjugate **12**.

The fully deprotected SAM-adenosine conjugate **12** could be obtained from either **9a** or **9b** (Scheme 5). Compound **9a** was successively treated with methylamine, ZnBr_2_ and cesium fluoride (CsF) to remove the benzoyl-, the Boc- and the TBS groups respectively to give **12** in 5% yield over three steps after HPLC purification. In comparison, a two-step strategy from **9b**, followed by HPLC purification, led to the formation of **12** in 4% yield. These results seem to indicate that in the pathway 2, the removal of the two Boc groups is less efficient than the two steps required for the deprotection of the benzoyl and Boc groups in the pathway 1 (Scheme 5).

**Scheme 6:**
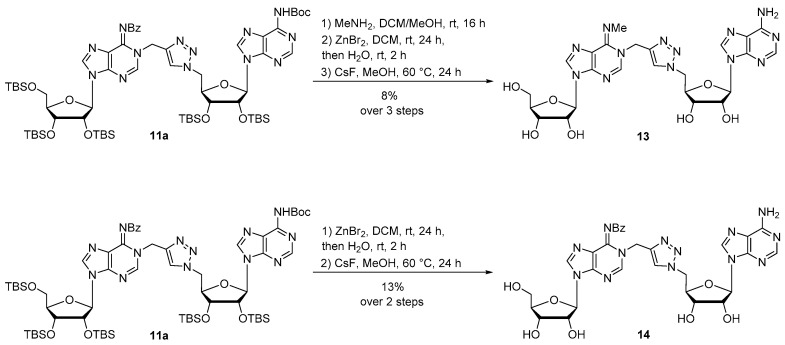
Access to SAM-adenosine conjugates **13** and **14**.

We applied the strategy used for the deprotection of compound **9a** to compound **11a**. Unfortunately, efforts to remove the protecting group of the exocyclic amine were unsuccessful. Indeed, using the same successive steps, we observed the formation of the *N*-methylated compound **13** in 8% yield as a mixture of *E* and *Z* imines (Scheme 6). Other attempts were conducted with bases such as ammonia or potassium carbonate which led to the recovery of the starting material in the first case and to degradation in the second one. As an alternative, we chose to remove only the Boc and the TBS groups in a two-step sequence allowing for the formation of SAM-adenosine conjugate **14** in 13% yield (Scheme 6).

**Scheme 7:**
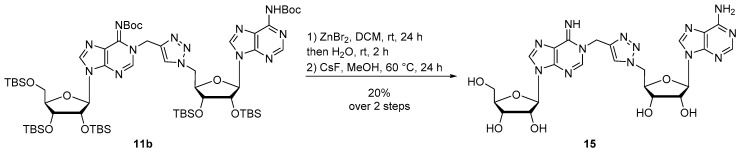
Access to SAM-adenosine conjugate **15**.

By contrast and to our delight, treating derivative **11b** with ZnBr_2_ and then CsF afforded the fully deprotected SAM-adenosine conjugate **15** in 20% yield over two steps (Scheme 7). Of note, deprotection of compounds **8a** and **10a** using methylamine and then CsF provided compounds **12** and **14**, respectively that could not be properly purified in these particular conditions.

### 2.2. Synthesis of SAM-Adenosine Conjugates without Protecting Groups

**Scheme 8:**
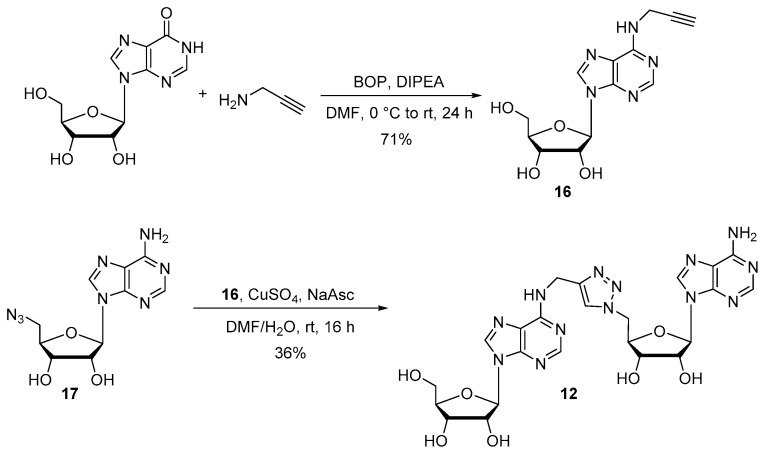
Access to SAM-adenosine conjugate **12**.

Since our syntheses require numerous steps of protection and deprotection, we reinvestigated the synthetic strategies to develop more efficient approaches to get the SAM-adenosine conjugates. We also sought to introduce regioselectively the propargyl group at the N-6 and N-1 positions. We first modified the synthesis of *N*^6^-conjugate **12**. Wan et al. previously reported the amination of unprotected inosine using primary amines in the presence of BOP and DIPEA [55]. Following this methodology, the propargyl group was introduced in one step at the N-6 position of adenosine leading to compound **16** in 71% yield (Scheme 8). 

The unprotected azido partner **17** was prepared following a two-step procedure in 36% yield [66,67]. Then, alkyne **16** and azide **17** were reacted in the presence of copper sulfate and sodium ascorbate in a DMF/H_2_O mixture to afford the expected SAM-adenosine conjugate **12** in 36% yield (Scheme 8).

**Scheme 9:**
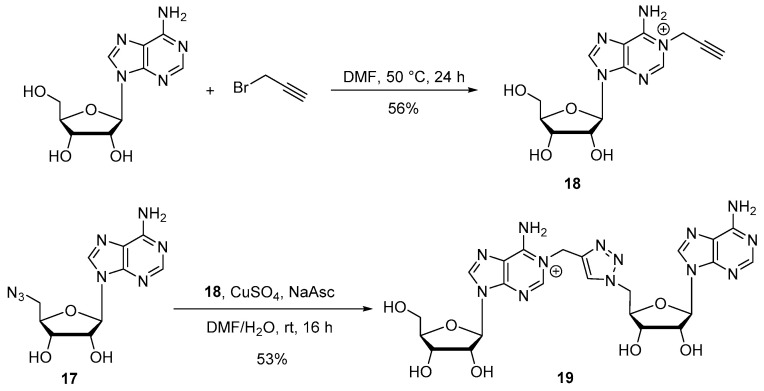
Access to SAM-adenosine conjugate **19**.

We next investigated the synthesis of 1-propargyladenosine **18** (Scheme 9). Adenosine is known to react with alkyl halides in polar solvents such as DMF or DMA at room temperature, to afford 1-*N*-alkylated compounds [40,51]. Under these conditions, adenosine was treated with an excess of propargyl bromide in DMF at 50 °C for 24 h and 1-*N*-propargylated adenosine **18** was obtained in 56% yield (Scheme 9). The synthesis of SAM-adenosine conjugate with a triazole linker connected at the N-1 position was then achieved through the CuAAC between **17** and **18**. The reaction was carried out in the presence of sodium ascorbate and copper sulfate to afford the expected conjugate **19** in 53% yield (Scheme 9). Compound **19** corresponds to the protonated and positively charged form of compound **15**. This was confirmed by ^1^H NMR spectra analysis for both compounds **15** and **19** (See the supporting file for NMR spectra of compounds **15** and **19**). The signal for 2-H (H2a) appears at 8.62 ppm for compound **19** while it is upfield at 8.22 ppm for compound **15** as expected [51].

## 3. Materials and Methods 

Reactions were carried out under argon atmosphere and solvents were dried using standard methods and distilled before use. DCM, Pyridine and DMF were dried over calcium hydride and THF over sodium and benzophenone. Unless otherwise specified, materials were purchased from commercial suppliers and used without further purification. TLC was performed using Merck commercial aluminum sheets coated with silica gel 60 F254 (Merck, Darmstadt, Germany). Compounds were detected by charring with 10% H_2_SO_4_ in ethanol followed by heating. Purification was performed by flash chromatography on silica gel (60 Å, 180–240 mesh; Merck, Darmstadt, Germany). Preparative HPLC was performed using a HPLC system with a reverse phase C-18 column (250 × 21.2 mm) using a solvent system consisting of H_2_O and CH_3_CN (linear gradient from 0:100 to 100:0 in 30 min) at a flow rate of 15 mL.min^−1^ and UV detection at 254 nm. The purity of final compounds (≥ 95%) was established by analytical HPLC, which was performed on Macherey Nagel C18 100-5 NUCLEOSIL column (25 × 4.6, 5 μm) with UV detection at 214 and 254 nm. NMR spectra were recorded on Bruker spectrometers (Avance II 500 and Avance III HD 4000) (Bruker Biospin, Fällanden, Switzerland). Chemical shifts (δ) are reported in parts per million (ppm) and referenced to the residual proton or carbon resonance of the solvents: CDCl_3_ (δ 7.26), MeOD (δ 3.31), D_2_O (δ 4.79) or (CD_3_)_2_SO (δ 2.50) for ^1^H and CDCl_3_ (δ 77.16), MeOD (δ 49.0) or (CD_3_)_2_SO (δ 39.52) for ^13^C. Signals were assigned using 1D (^1^H and ^13^C) and 2D (HSQC, COSY and HMBC) experiments. NMR coupling constants (*J*) are reported in Hertz (Hz) and splitting patterns are indicated as follows: s (singlet), bs (broad singlet), d (doublet), t (triplet), q (quartet), dd (doublet of doublet), m (multiplet). High-resolution mass spectroscopy (HRMS) was recorded with an ion trap mass analyzer under electrospray ionization (ESI) in the negative or positive ionization detection mode, using Thermo Scientific LTQ Orbitrap XL (Thermo Scientific, Illkirch, France.

Compounds **2a** and **3a**: The trisilylated adenosine **1a** (1.59 g, 2.22 mmol) and propargyl bromide 80% in toluene (674 μL, 8.88 mmol) were dissolved in DCM (50 mL) and tetrabutylammonium hydroxyde (1.78 g, 2.22 mmol) and 1M aqueous NaOH (22.2 mL) were added to the solution. After vigorous stirring at room temperature for 1 h, the reaction mixture was diluted in DCM and washed with brine, dried over MgSO_4_, and the solvent was removed under reduced pressure. The residue was purified by silica gel chromatography (eluent: Cyclohexane/EtOAc 9:1 then 7:3) to provide the desired compounds as white foams (946 mg, 57% for **2a** and 475 mg, 28% for **3a**). **2a**: ^1^H NMR (500 MHz, CDCl_3_): δ 8.49 (s, 1H, H2), 8.14 (s, 1H, H8), 7.39–7.37 (m, 2H, H^Bz^), 7.18–7.15 (m, 1H, H^Bz^), 7.06–7.03 (m, 2H, H^Bz^), 5.95 (d, *J* = 5.9 Hz, 1H, H1′), 5.7 (d, *J* = 4.9 Hz, 2H, CH_2_N), 4.54–4.52 (m, 1H, H2′), 4.17–4.15 (m, 1H, H3′), 4.03–4.01 (m, 1H, H4′), 3.89 (dd, *J* = 4.4, 11.4 Hz, 1H, H5′), 3.68 (dd, *J* = 2.8, 11.2 Hz, 1H, H5′), 2.01 (t, *J* = 4.7 Hz, 1H, C≡CH), 0.85 (s, 9H, *t*Bu^TBS^), 0.83 (s, 9H, *t*Bu^TBS^), 0.64 (s, 9H, *t*Bu^TBS^), 0.02 (s, 3H, Me^TBS^), 0.01 (s, 3H, Me^TBS^), 0.00 (s, 6H, 2 Me^TBS^), −0.19 (s, 3H, Me^TBS^), −0.51 (s, 3H, Me^TBS^). ^13^C NMR (126 MHz, CDCl_3_): δ 171.4 (C=O), 153.2 (C6), 152.8 (C4), 151.9 (C2), 142.8 (C8), 135.9 (Cq^Bz^), 130.9 (C^Bz^), 128.9 (2C, C^Bz^), 127.9 (2C, C^Bz^), 127.0 (C5), 88.2 (C1′), 86.2 (C4′), 79.0 (C≡CH), 75.8 (C2′), 72.4 (C3′), 71.7 (C≡CH), 62.8 (C5′), 37.5 (CH_2_N), 26.1 (3C, *t*Bu^TBS^), 25.9 (3C, *t*Bu^TBS^), 25.7 (3C, *t*Bu^TBS^), 18.6 (Cq^TBS^), 18.1 (Cq^TBS^), 17.9 (Cq^TBS^), −4.3 (Me^TBS^), −4.4 (Me^TBS^), −4.5 (Me^TBS^), −5.0 (Me^TBS^), −5.2 (Me^TBS^), −5.3 (Me^TBS^). HRMS (ESI) *m*/*z*: calcd for C_38_H_61_N_5_NaO_5_Si_3_ [M + Na]^+^: 774.3878; found: 774.3904. **3a**: ^1^H NMR (500 MHz, CDCl_3_): δ 8.24 (s, 1H, H2), 8.16–8.15 (m, 2H, H^Bz^), 7.88 (s, 1H, H8), 7.48–7.45 (m, 1H, H^Bz^), 7.40–7.37 (m, 2H, H^Bz^), 5.90 (d, *J* = 5.0 Hz, 1H, H1′), 4.97 (t, *J* = 2.8 Hz, 2H, CH_2_N), 4.48–4.47 (m, 1H, H2′), 4.26–4.24 (m, 1H, H3′), 4.08–4.06 (m, 1H, H4′), 3.88 (dd, *J* = 4.0, 11.3 Hz, 1H, H5′), 3.73 (dd, *J* = 3.4, 11.4 Hz, 1H, H5′), 2.59 (t, *J* = 2.5 Hz, 1H, C≡CH), 0.92 (s, 9H, *t*Bu^TBS^), 0.89 (s, 9H, *t*Bu^TBS^), 0.81 (s, 9H, *t*Bu^TBS^), 0.09 (s, 3H, Me^TBS^), 0.08 (s, 3H, Me^TBS^), 0.06 (s, 3H, Me^TBS^), 0.05 (s, 3H, Me^TBS^), −0.03 (s, 3H, Me^TBS^), −0.19 (s, 3H, Me^TBS^). ^13^C NMR (126 MHz, CDCl_3_): δ 177.2 (C=O), 146.1 (C6), 145.4 (C2), 145.1 (C4), 138.8 (C8), 135.8 (Cq^Bz^), 132.0 (C^Bz^), 129.9 (2C, C^Bz^), 128.1 (2C, C^Bz^), 122.3 (C5), 88.2 (C1′), 85.4 (C4′), 76.7 (C≡CH), 76.1 (C2′), 75.9 (C≡CH), 72.0 (C3′), 62.7 (C5′), 37.8 (CH_2_N), 26.1 (3C, *t*Bu^TBS^), 25.9 (3C, *t*Bu^TBS^), 25.8 (3C, *t*Bu^TBS^), 18.6 (Cq^TBS^), 18.2 (Cq^TBS^), 18.0 (Cq^TBS^), −4.2 (Me^TBS^), −4.4 (Me^TBS^), −4.5 (Me^TBS^), −4.8 (Me^TBS^), −5.2 (Me^TBS^), −5.3 (Me^TBS^). HRMS (ESI) *m*/*z*: calcd for C_38_H_61_N_5_NaO_5_Si_3_ [M + Na]^+^: 774.3878; found: 774.3911.

Compounds **2b** and **3b**: To a stirred solution of compound **1b** (1.5 g, 2.11 mmol) in DCM (47.5 mL) was added propargyl bromide (80% in toluene) (0.795 mL, 8.44 mmol), tetrabutylammonium hydroxide (1.68 g, 1.68 mmol) and 1M aqueous NaOH (21.1 mL). The reaction mixture was stirred at room temperature for 1 h. The reaction mixture was dissolved in DCM and washed with brine. The organic layer was dried over MgSO_4_, concentrated in vacuo and purified on silica gel chromatography (eluent: cyclohexane/EtOAc 9:1 then 7:3) to afford the desired compounds as white foams (1.2 g, 76% for **2b** and 0.251 g, 16% for **3b**). **2b**: ^1^H NMR (500 MHz, CDCl_3_): δ 8.77 (s, 1H, H2), 8.41 (s, 1H, H8), 6.10 (d, *J* = 4.9 Hz, 1H, H1′), 4.80 (d, *J* = 2.4 Hz, 2H, CH_2_N), 4.64 (t, *J* = 4.6 Hz, 1H, H2′), 4.33 (t, *J* = 4.0 Hz, 1H, H3′), 4.14 (q, *J* = 3.8 Hz, 1H, H4′), 4.04 (dd, *J* = 11.4, 4.1 Hz, 1H, H5′), 3.80 (dd, *J* = 11.4, 2.8 Hz, 1H, H5′), 2.16 (t, *J* = 2.4 Hz, 1H, C≡CH), 1.48 (s, 9H, *t*Bu^Boc^), 0.96 (s, 9H, *t*Bu^TBS^), 0.93 (s, 9H, *t*Bu^TBS^), 0.80 (s, 9H, *t*Bu^TBS^), 0.15 (s, 3H, Me^TBS^), 0.14 (s, 3H, Me^TBS^), 0.10 (s, 3H, Me^TBS^), 0.09 (s, 3H, Me^TBS^), −0.04 (s, 3H, Me^TBS^), −0.22 (s, 3H, Me^TBS^). ^13^C NMR (126 MHz, CDCl_3_): δ 152.7 (C6), 152.5 (C4), 152.4 (C=O), 151.8 (C2), 142.2 (C8), 127.60 (C5), 88.7 (C1′), 85.5 (C4′), 82.9 (Cq^Boc^), 79.9 (C≡CH), 76.1 (C2′), 72.0 (C3′), 71.2 (C≡CH), 62.6 (C5′), 37.3 (CH_2_N), 28.1 (3C, *t*Bu^Boc^), 26.3 (3C, *t*Bu^TBS^), 26.0 (3C, *t*Bu^TBS^), 25.8 (3C, *t*Bu^TBS^), 18.7 (Cq^TBS^), 18.2 (Cq^TBS^), 18.0 (Cq^TBS^), −4.2 (Me^TBS^), −4.5 (Me^TBS^), −4.6 (Me^TBS^), −4.8 (Me^TBS^), −5.2 (Me^TBS^), −5.2 (Me^TBS^). HRMS (ESI) *m*/*z*: calcd for C_36_H_66_N_5_O_6_Si_3_ [M + H]^+^: 748,4315; found 748.4304. **3b**: ^1^H NMR (500 MHz, CDCl_3_): δ 8.22 (s, 1H, H2), 8.12 (s, 1H, H8), 5.91 (d, *J* = 3.3 Hz, 1H, H1′), 4.83 (d, *J* = 2.7 Hz, 2H, CH_2_N), 4.36–4.30 (m, 2H, H2′, H3′), 4.11–4.08 (m, 1H, H4′), 3.99 (dd, *J* = 11.6, 3.4 Hz, 1H, H5′), 3.78 (dd, *J* = 11.6, 2.5 Hz, 1H, H5′), 2.56 (t, *J* = 2.6 Hz, 1H, C≡CH), 1.60 (s, 9H, *t*Bu^Boc^), 0.94 (s, 9H, *t*Bu^TBS^), 0.92 (s, 9H, *t*Bu^TBS^), 0.86 (s, 9H, *t*Bu^TBS^), 0.12 (s, 3H, Me^TBS^), 0.11 (s, 3H, Me^TBS^), 0.10 (s, 3H, Me^TBS^), 0.08 (s, 3H, Me^TBS^), 0.02 (s, 3H, Me^TBS^), −0.04 (s, 3H, Me^TBS^). ^13^C NMR (126 MHz, CDCl_3_): δ 161.0 (C=O), 147.1 (C6), 145.1 (C2), 144.2 (C4), 138.2 (C8), 122.0 (C5), 88.7 (C1′), 84.5 (C4′), 80.9 (Cq^Boc^), 76.8 (C≡CH), 76.7 (C2′), 76.4 (C≡CH), 70.9 (C3′), 61.9 (C5′), 37.0 (CH_2_N), 28.3 (3C, *t*Bu^Boc^), 26.3 (3C, *t*Bu^TBS^), 26.0 (3C, *t*Bu^TBS^), 25.9 (3C, *t*Bu^TBS^), 18.7 (Cq^TBS^), 18.2 (Cq^TBS^), 18.1 (Cq^TBS^), −4.1 (Me^TBS^), −4.6 (2C, Me^TBS^), −4.7 (Me^TBS^), −5.2 (Me^TBS^), −5.3 (Me^TBS^). HRMS (ESI) *m*/*z*: calcd for C_36_H_66_N_5_O_6_Si_3_ [M + H]^+^: 748.4315; found 748.4309.

Compound **4**: To a stirred solution of compound **1a** (1.42 g, 1.97 mmol) in THF (20 mL) at 0 °C, was added dropwise an aqueous solution of TFA (1:1, 7.55 mL, 98.72 mmol). The solution was stirred at room temperature for 1.5 h. The reaction mixture was neutralized with saturated aqueous NaHCO_3_ solution and dissolved in EtOAc. The organic layer was washed with brine, dried over MgSO_4_, filtered and concentrated in vacuo. The residue was purified on silica gel chromatography (eluent: Cyclohexane/EtOAc 6:4) to afford compound **4** as a white foam (1.02 g, 86%). ^1^H NMR (500 MHz, CDCl_3_): δ 8.83 (s, 1H, H2), 8.13 (s, 1H, H8), 8.07–8.03 (m, 2H, H^Bz^), 7.65–7.60 (m, 1H, H^Bz^), 7.56–7.51 (m, 2H, H^Bz^), 5.88 (d, *J* = 7.6 Hz, 1H, H1′), 5.03 (dd, *J* = 7.6, 4.5 Hz, 1H, H2′), 4.35 (d, *J* = 4.5 Hz, 1H, H3′), 4.20 (d, *J* = 1.7 Hz, 1H, H4′), 3.98 (dd, *J* = 13.1, 1.8 Hz, 1H, H5′), 3.74 (dd, *J* = 13.0, 1.6 Hz, 1H, H5′), 0.96 (s, 9H, *t*Bu^TBS^), 0.75 (s, 9H, *t*Bu^TBS^), 0.14 (s, 3H, Me^TBS^), 0.13 (s, 3H, Me^TBS^), −0.12 (s, 3H, Me^TBS^), −0.60 (s, 3H, Me^TBS^). ^13^C NMR (126 MHz, CDCl_3_): δ 164.4 (C=O), 152.4 (C6), 150.6 (C2), 150.5 (C4), 143.4 (C8), 133.7 (Cq^Bz^), 133.1 (C^Bz^), 129.1 (2C, C^Bz^), 127.9 (2C, C^Bz^), 124.5 (C5), 91.4 (C1′), 89.7 (C4′), 74.2 (C2′), 74.0 (C3′), 63.1 (C5′), 25.9 (3C, *t*Bu^TBS^), 25.8 (3C, *t*Bu^TBS^), 18.2 (Cq^TBS^), 17.9 (Cq^TBS^), −4.4 (Me^TBS^), −4.4 (Me^TBS^), −4.5 (Me^TBS^), −5.7 (Me^TBS^). HRMS (ESI) *m*/*z*: calcd for C_29_H_46_N_5_O_5_Si_2_ [M + H]^+^: 600.3032; found: 600.3022.

Compound **5**: Methanesulfonyl chloride (0.26 mL, 3.33 mmol) in pyridine (10 mL) was added dropwise to a solution of compound **4** (1 g, 1.66 mmol) in pyridine (7 mL) at 0 °C. The reaction mixture was stirred at room temperature for 16 h. The reaction was quenched by addition of water and then diluted in DCM. The organic layer was washed with a saturated solution of NaHCO_3_ and brine. The combined organic layer was dried over MgSO_4_ and concentrated to dryness. The residue was purified on silica gel chromatography (eluent: Cyclohexane/EtOAc 6:4) and compound **5** was isolated as a white foam (0.92 g, 82%). ^1^H NMR (500 MHz, CDCl_3_): δ 8.82 (s, 1H, H2), 8.20 (s, 1H, H8), 8.04 (d, *J* = 7.3 Hz, 2H, H^Bz^), 7.62 (t, *J* = 7.4 Hz, 1H, H^Bz^), 7.54 (t, *J* = 7.6 Hz, 2H, H^Bz^), 6.00 (d, *J* = 4.8 Hz, 1H, H1′), 4.99 (t, *J* = 4.4 Hz, 1H, H2′), 4.62 (dd, *J* = 11.2, 4.1 Hz, 1H, H5′), 4.50 (dd, *J* = 11.2, 4.6 Hz, 1H, H5′), 4.40 – 4.35 (m, 2H, H3′, H4′), 3.03 (s, 3H, CH_3_), 0.95 (s, 9H, *t*Bu^TBS^), 0.82 (s, 9H, *t*Bu^TBS^), 0.15 (s, 3H, Me^TBS^), 0.13 (s, 3H, Me^TBS^), 0.00 (s, 3H, Me^TBS^), −0.20 (s, 3H, Me^TBS^). ^13^C NMR (126 MHz, CDCl_3_): δ 168.3 (C=O), 152.8 (C6), 151.5 (C2), 149.9 (C4), 142.5 (C8), 134.8 (Cq^Bz^), 132.9 (C^Bz^), 129.0 (2C, C^Bz^), 127.9 (2C, C^Bz^),123.9 (C5), 89.9 (C1′), 82.4 (C4′), 74.3 (C2′), 72.1 (C3′), 67.9 (C5′), 37.8 (CH_3_), 25.9 (3C, *t*Bu^TBS^), 25.8 (3C, *t*Bu^TBS^), 18.2 (Cq^TBS^), 18.00 (Cq^TBS^), −4.3 (Me^TBS^), −4.5 (Me^TBS^), −4.7 (Me^TBS^), −4.8 (Me^TBS^). HRMS (ESI) *m*/*z*: calcd for C_30_H_48_N_5_O_7_SSi_2_ [M + H]^+^: 678.2807; found: 678.2788.

Compound **6**: To a stirred solution of compound **5** (920 mg, 1.35 mmol) in DMF (7 mL) was added sodium azide (264 mg, 4.06 mmol) and the mixture was heated at 70 °C for 4 h. The reaction mixture was cooled to room temperature and was dissolved in EtOAc and washed with brine. The organic layer was dried over MgSO_4_, concentrated in vacuo and purified on silica gel chromatography (eluent: Cyclohexane/EtOAc 8:2) to afford compound **6** as a white foam (770 mg, 92%). ^1^H NMR (500 MHz, CDCl_3_): δ 8.80 (s, 1H, H2), 8.28 (s, 1H, H8), 8.03 (d, *J* = 7.3, 2H, H^Bz^), 7.60 (t, *J* = 7.3, 1H, H^Bz^), 7.52 (t, *J* = 7.8, 2H, H^Bz^), 5.98 (d, *J* = 4.0 Hz, 1H, H1′), 4.88 (t, *J* = 4.1 Hz, 1H, H2′), 4.31 (t, *J* = 4.6 Hz, 1H, H3′), 4.23 (q, *J* = 4.6 Hz, 1H, H4′), 3.80–3.67 (m, 2H, H5′), 0.94 (s, 9H, *t*Bu^TBS^), 0.84 (s, 9H, *t*Bu^TBS^), 0.12 (s, 3H, Me^TBS^), 0.11 (s, 3H, Me^TBS^), −0.01 (s, 3H, Me^TBS^), −0.12 (s, 3H, Me^TBS^). ^13^C NMR (126 MHz, CDCl_3_): δ 164.6 (C=O), 152.8 (C6), 151.5 (C2), 149.8 (C4), 142.4 (C8), 133.8 (Cq^Bz^), 132.9 (C^Bz^), 129.0 (2C, C^Bz^), 127.9 (2C, C^Bz^), 123.90 (C5), 90.1 (C1′), 82.8 (C4′), 74.8 (C2′), 72.3 (C3′), 51.6 (C5′), 25.9 (3C, *t*Bu^TBS^), 25.8 (3C, *t*Bu^TBS^), 18.2 (Cq^TBS^), 18.0 (Cq^TBS^), −4.2 (Me^TBS^), −4.5 (Me^TBS^), −4.6 (Me^TBS^), −4.7 (Me^TBS^). HRMS (ESI) *m*/*z*: calcd for C_29_H_45_N_8_O_4_Si_2_ [M + H]^+^: 625.3096; found 625.3088.

General procedure A for CuAAC reaction: To a solution of alkyne (1 eq) in THF (13 mL/mmol), were successively added azido compound **6** or **7** (1.2 eq), CuSO_4_ (0.3 eq, in water 3 mL/mmol) and sodium ascorbate (0.6 eq, in water 3 mL/mmol). The heterogeneous mixture was stirred at room temperature for 16 h. EtOAc was added and the organic layer was washed with brine, dried over MgSO_4_, and concentrated in vacuo. The crude was purified by flash chromatography to afford the desired compounds.

Compound **8a**: Following the general procedure A for CuAAC, starting from alkyne **2a** (200 mg, 0.26 mmol) and azido compound **6** (194 mg, 0.31 mmol) and using Cyclohexane/EtOAc 6:4 as eluent for flash chromatography purification, compound **8a** was obtained as a white foam (245 mg, 68%). ^1^H NMR (500 MHz, CDCl_3_): δ 8.84 (s, 1H, H2a or H2b), 8.52 (s, 1H, H2a or H2b), 8.21 (s, 1H, H8a or H8b), 8.02 (bs, 3H, H8a or H8b and H^Bz^), 7.81 (s, 1H, H^Triazole^), 7.60 (bs, 1H, H^Bz^), 7.53 (bs, 2H, H^Bz^), 7.42 (d, *J* = 7.1 Hz, 2H, H^Bz^), 7.22 (t, *J* = 7.4 Hz, 1H, H^Bz^), 7.10 (t, *J* = 7.6 Hz, 2H, H^Bz^), 5.99 (d, *J* = 5.6 Hz, 1H, H1′a), 5.90 (bs, 1H, H1′b), 5.64 (s, 2H, CH_2_N), 5.07 (bs, 1H, H2′b), 4.89–4.81 (m, 1H, H5′b), 4.65–4.58 (m, 2H, H5′b and H2′a), 4.43 (bs, 1H, H3′b), 4.36 (bs, 1H, H4′b), 4.26–4.24 (m, 1H, H3′a), 4.11–4.08 (m, 1H, H4′a), 3.97 (dd, *J* = 11.3, 4.3 Hz, 1H, H5′a), 3.76 (dd, *J* = 11.3, 2.9 Hz, 1H, H5′a), 0.94 (s, 9H, *t*Bu^TBS^), 0.92 (s, 9H, *t*Bu^TBS^), 0.89 (s, 9H, *t*Bu^TBS^), 0.76 (s, 9H, *t*Bu^TBS^), 0.74 (s, 9H, *t*Bu^TBS^), 0.11 (s, 3H, Me^TBS^), 0.11 (s, 3H, Me^TBS^), 0.09 (s, 3H, Me^TBS^), 0.09 (s, 3H, Me^TBS^), 0.04 (s, 3H, Me^TBS^), −0.05 (s, 3H, Me^TBS^), −0.08 (s, 3H, Me^TBS^), −0.11 (s, 3H, Me^TBS^), −0.37 (s, 3H, Me^TBS^), −0.42 (s, 3H, Me^TBS^). ^13^C NMR (126 MHz, CDCl_3_): δ 172.2 (2C, C=O), 164.5 (Cq), 153.7 (Cq), 152.8 (Cq), 152.0 (C2a or C2b), 151.5 (Cq), 150.1 (C2a or C2b), 144.7 (Cq^Triazole^), 143.2 (C8a or C8b), 143.0 (C8a or C8b), 136.0 (Cq^Bz^) 133.9 (Cq^Bz^) 132.9 (C^Bz^), 130.9 (C^Bz^), 129.1 (2C, C^Bz^), 129.0 (2C, C^Bz^), 127.9 (4C, C^Bz^), 127.30 (Cq), 124.9 (CH^Triazole^), 124.2 (Cq), 90.1 (C1′b), 88.4 (C1′a), 86.0 (C4′a), 84.1 (C4′b), 75.8 (C2′a), 73.4 (C3′b), 73.3 (C2′a), 72.3 (C3′a), 62.8 (C5′a), 51.7 (C5′b), 44.0 (CH_2_N), 26.2 (3C, *t*Bu^TBS^), 26.0 (3C, *t*Bu^TBS^), 25.9 (3C, *t*Bu^TBS^), 25.8 (6C, *t*Bu^TBS^), 18.6 (Cq^TBS^), 18.2 (Cq^TBS^), 18.1 (Cq^TBS^), 17.9 (Cq^TBS^), 17.8 (Cq^TBS^), −4.3 (Me^TBS^), −4.5 (3C, Me^TBS^), −4.6 (Me^TBS^), −4.7 (Me^TBS^), −4.9 (Me^TBS^), −5.0 (Me^TBS^), −5.2 (Me^TBS^), −5.3 (Me^TBS^). HRMS (ESI) *m*/*z*: calcd for C_67_H_106_N_13_O_9_Si_5_ [M + H]^+^: 1376.7077; found 1376.7056.

Compound **9a**: Following the general procedure A for CuAAC, starting from alkyne **2a** (578 mg, 0.77 mmol) and azido compound **7** (277 mg, 0.38 mmol) and using Cyclohexane/EtOAc 7:3 as eluent for flash chromatography purification, compound **9a** was obtained as a white foam (384 mg, 60%). ^1^H NMR (500 MHz, CDCl_3_): δ 8.75 (s, 1H, H8 or H2), 8.51 (s, 1H, H8 or H2), 8.19 (s, 1H, H8 or H2), 8.04 (bs, 1H, NH), 7.92 (bs, 1H, H8 or H2), 7.80 (s, 1H, H^Triazole^), 7.42–7.40 (m, 2H, H^Bz^), 7.23–7.20 (m, 1H, H^Bz^), 7.11–7.08 (m, 2H, H^Bz^), 5.98 (d, *J* = 5.6 Hz, 1H, H1′a), 5.86 (d, *J* = 6.2 Hz, 1H, H1′b), 5.63 (s, 2H, CH_2_N), 5.04–5.02 (m, 1H, H2′b), 4.84 (dd, *J* = 6.3, 14.2 Hz, 1H, H5′b), 4.61–4.57 (m, 2H, H5′b and H2′a), 4.39–4.38 (m, 1H, H3′b), 4.35–4.32 (m, 1H, H4′b), 4.25–4.23 (m, 1H, H3′a), 4.10–4.08 (m, 1H, H4′a), 3.96 (dd, *J* = 4.4, 11.3 Hz, 1H, H5′a), 3.75 (dd, *J* = 3.0, 11.4 Hz, 1H, H5′a), 1.56 (s, 9H, *t*Bu^Boc^), 0.93 (s, 9H, *t*Bu^TBS^), 0.91 (s, 9H, *t*Bu^TBS^), 0.87 (s, 9H, *t*Bu^TBS^), 0.73 (s, 18H, *t*Bu^TBS^), 0.11 (s, 3H, Me^TBS^), 0.10 (s, 3H, Me^TBS^), 0.08 (s, 6H, Me^TBS^), 0.02 (s, 3H, Me^TBS^), −0.08 (s, 3H, Me^TBS^), −0.11 (s, 6H, Me^TBS^), −0.43 (s, 6H, Me^TBS^). ^13^C NMR (126 MHz, CDCl_3_): δ 172.1 (C=O), 153.7 (C2 or C8), 153.1 (Cq), 152.8 (Cq), 152.0 (C2 or C8), 150.6 (Cq), 150.4 (Cq), 149.6 (Cq), 144.7 (Cq), 142.9 (C2 or C8), 142.6 (C2 or C8), 135.9 (Cq), 130.9 (C^Bz^), 129.0 (2C, C^Bz^), 127.9 (2C, C^Bz^), 127.3 (Cq^Bz^), 124.9 (CH^Triazole^), 122.9 (Cq), 89.8 (C1′b), 88.3 (C1′a), 86.0 (C4′a), 84.2 (C4′b), 82.4 (Cq^Boc^), 75.8 (C2′a), 73.4 (C3′b), 73.1 (C2′b), 72.2 (C3′a), 62.7 (C5′a), 51.7 (C5′b), 44.0 (CH_2_N), 28.3 (3C, *t*Bu^Boc^), 26.2 (3C, *t*Bu^TBS^), 25.9 (3C, *t*Bu^TBS^), 25.8 (3C, *t*Bu^TBS^), 25.7 (6C, *t*Bu^TBS^), 18.6 (Cq^TBS^), 18.2 (Cq^TBS^), 18.1 (Cq^TBS^), 17.9 (2C, Cq^TBS^), −4.3 (Me^TBS^), −4.5 (3C, 3 Me^TBS^), −4.6 (Me^TBS^), −4.8 (Me^TBS^), −5.0 (Me^TBS^), −5.1 (Me^TBS^), −5.2 (Me^TBS^), −5.3 (Me^TBS^). HRMS (ESI) *m*/*z*: calcd for C_65_H_108_N_13_O_10_Si_5_ [M − H]^−^: 1370.7188; found: 1370.7132.

Compound **9b**: Following the general procedure A for CuAAC, starting from alkyne **2b** (50 mg, 0.067 mmol) and azido compound **7** (49 mg, 0.08 mmol) and using Cyclohexane/EtOAc 7:3 as eluent for flash chromatography purification, compound **9b** was obtained as a white foam (68 mg, 74%). ^1^H NMR (500 MHz, CDCl_3_): δ 8.74 (s, 1H, H2b), 8.70 (s, 1H, H2a), 8.38 (s, 1H, H8a), 8.00 (s, 1H, NH), 7.90 (s, 1H, H8b), 7.70 (s, 1H, H^Triazole^), 6.07 (d, *J* = 4.7 Hz, 1H, H1′a), 5.86 (d, *J* = 6.2 Hz, 1H, H1′b), 5.30 (s, 2H, CH_2_N), 5.10 (dd, *J* = 6.2, 4.3 Hz, 1H, H2′b), 4.89 (dd, *J* = 14.3, 6.3 Hz, 1H, H5′b), 4.65–4.58 (m, 2H, H5′b, H2′a), 4.41 (dd, *J* = 4.3, 2.4 Hz, 1H, H3′b), 4.36–4.31 (m, 2H, H4′b, H3′a), 4.15–4.12 (m, 1H, H4′a), 4.03 (dd, *J* = 11.4, 4.1 Hz, 1H, H5′a), 3.79 (dd, *J* = 11.4, 2.9 Hz, 1H, H5′a), 1.57 (s, 9H, *t*Bu^Boc^), 1.38 (s, 9H, *t*Bu^Boc^), 0.95 (s, 9H, *t*Bu^TBS^), 0.93 (s, 9H, *t*Bu^TBS^), 0.88 (s, 9H, *t*Bu^TBS^), 0.80 (s, 9H, *t*Bu^TBS^), 0.74 (s, 9H, *t*Bu^TBS^), 0.13 (s, 3H, Me^TBS^), 0.12 (s, 3H, Me^TBS^), 0.10 (s, 3H, Me^TBS^), 0.09 (s, 3H, Me^TBS^), 0.04 (s, 3H, Me^TBS^), −0.04 (s, 3H, Me^TBS^), −0.06 (s, 3H, Me^TBS^), −0.10 (s, 3H, Me^TBS^), −0.21 (s, 3H, Me^TBS^), −0.42 (s, 3H, Me^TBS^). ^13^C NMR (126 MHz, CDCl_3_): δ 153.4 (C2b), 153.1 (Cq), 152.9 (Cq), 152.5 (Cq), 151.9 (C2a), 150.6 (Cq), 150.4 (C=O), 149.6 (C=O), 145.8 (Cq^Triazole^), 142.6 (C8b), 142.2 (C8a), 127.7 (Cq), 124.1 (CH^Triazole^), 122.9 (Cq), 90.1 (C1′b), 88.8 (C1′a), 85.3 (C4′a), 84.4 (C4′b), 82.5 (2C, Cq^Boc^), 75.9 (C2′a), 73.5 (C3′b), 73.1 (C2′b), 71.8 (C3′a), 62.5 (C5′a), 51.8 (C5′b), 43.4 (CH_2_N), 28.3 (3C, *t*Bu^Boc^), 28.0 (3C, *t*Bu^Boc^), 26.2 (3C, *t*Bu^TBS^), 26.0 (3C, *t*Bu^TBS^), 25.9 (3C, *t*Bu^TBS^), 25.8 (3C, *t*Bu^TBS^), 25.7 (3C, *t*Bu^TBS^), 18.7 (Cq^TBS^), 18.2 (Cq^TBS^), 18.1 (Cq^TBS^), 18.0 (Cq^TBS^), 17.9 (Cq^TBS^), −4.2 (Me^TBS^), −4.4 (Me^TBS^), −4.5 (Me^TBS^), −4.5 (Me^TBS^), −4.6 (Me^TBS^), −4.7 (Me^TBS^), −4.8 (Me^TBS^), −5.1 (Me^TBS^), −5.2 (Me^TBS^), −5.3 (Me^TBS^). HRMS (ESI) *m*/*z*: calcd for C_63_H_114_N_13_O_11_Si_5_ [M + H]^+^: 1368.7601; found 1368.7601.

Compound **10a**: Following the general procedure A for CuAAC, starting from alkyne **3a** (200 mg, 0.26 mmol) and azido compound **6** (194 mg, 0.31 mmol) and using Cyclohexane/EtOAc 5:5 as eluent for flash chromatography purification, compound **10a** was obtained as a white foam (258 mg, 72%). ^1^H NMR (500 MHz, CDCl_3_): δ 8.70 (s, 1H, H2b), 8.26 (s, 1H, H2a), 8.05 (d, *J* = 7.4 Hz, 2H, H^Bz^), 7.95 (d, *J* = 7.2 Hz, 2H, H^Bz^), 7.79 (s, 2H, H8a and H8b), 7.74 (s, 1H, H^Triazole^), 7.62 (t, *J* = 7.3 Hz, 1H, H^Bz^), 7.54 (t, *J* = 7.5 Hz, 2H, H^Bz^), 7.40 (t, *J* = 7.3 Hz, 1H, H^Bz^), 7.31 (t, *J* = 7.6 Hz, 2H, H^Bz^), 5.85–5.80 (m, 2H, H1′a and H1′b), 5.43–5.31 (m, 2H, CH_2_N), 5.13–5.07 (m, 1H, H2′b), 4.85 (dd, *J* = 14.2, 4.8 Hz, 1H, H5′b), 4.73 (dd, *J* = 14.2, 7.2 Hz, 1H, H5′b), 4.50 (t, *J* = 3.8 Hz, 1H, H3′b), 4.44 (t, *J* = 4.4 Hz, 1H, H2′a), 4.40–4.36 (m, 1H, H4′b), 4.23 (t, *J* = 4.3 Hz, 1H, H3′a), 4.05 (q, *J* = 3.9 Hz, 1H, H4′a), 3.86 (dd, *J* = 11.4, 4.1 Hz, 1H, H5′a), 3.69 (dd, *J* = 11.4, 3.4 Hz, 1H, H5′a), 0.91 (s, 9H, *t*Bu^TBS^), 0.90 (s, 9H, *t*Bu^TBS^), 0.86 (s, 9H, *t*Bu^TBS^), 0.80 (s, 9H, *t*Bu^TBS^), 0.79 (s, 9H, *t*Bu^TBS^), 0.10 (s, 3H, Me^TBS^), 0.07 (s, 3H, Me^TBS^), 0.06 (s, 3H, Me^TBS^), 0.05 (s, 3H, Me^TBS^), 0.04 (s, 3H, Me^TBS^), 0.03 (s, 3H, Me^TBS^), −0.04 (s, 3H, Me^TBS^), −0.06 (s, 3H, Me^TBS^), −0.19 (s, 3H, Me^TBS^), −0.34 (s, 3H, Me^TBS^). ^13^C NMR (126 MHz, CDCl_3_): δ 177.1 (C=O), 164.7 (C=O), 152.7 (C2b), 151.2 (Cq), 150.1 (Cq), 146.7 (C2a), 146.6 (Cq), 145.2 (Cq), 143.1 (C8b), 141.9 (Cq^Triazole^), 138.8 (C8a), 135.8 (Cq^Bz^), 133.8 (Cq^Bz^), 133.0 (C^Bz^), 131.9 (C^Bz^), 129.8 (2C, C^Bz^), 129.0 (2C, C^Bz^), 128.1 (4C, C^Bz^), 125.6 (CH^Triazole^), 124.2 (Cq), 122.5 (Cq), 90.5 (C1′b), 88.5 (C1′a), 85.1 (C4′a), 83.5 (C4′b), 76.0 (C2′a), 73.5 (C3′b), 73.4 (C2′b), 71.7 (C3′a), 62.6 (C5′a), 51.8 (C5′b), 43.7 (CH_2_N), 26.2 (3C, *t*Bu^TBS^), 26.0 (3C, *t*Bu^TBS^), 25.9 (3C, *t*Bu^TBS^), 25.8 (3C, *t*Bu^TBS^), 25.80 (3C, *t*Bu^TBS^), 18.6 (Cq^TBS^), 18.2 (Cq^TBS^), 18.1 (Cq^TBS^), 18.0 (2C, Cq^TBS^), −4.2 (Me^TBS^), −4.3 (Me^TBS^), −4.4 (Me^TBS^), −4.5 (Me^TBS^), −4.6 (2C Me^TBS^), −4.7 (Me^TBS^), −4.9 (Me^TBS^), −5.2 (Me^TBS^), −5.3 (Me^TBS^). HRMS (ESI) m/z: calcd for C_67_H_106_N_13_O_9_Si_5_ [M + H]^+^: 1376.7077; found 1376.7060.

Compound **11a**: Following the general procedure A for CuAAC, starting from alkyne **3a** (45 mg, 0.059 mmol) and azido compound **7** (19 mg, 0.03 mmol) and using Cyclohexane/EtOAc 7:3 as eluent for flash chromatography purification, compound **11a** was obtained as a white foam (37 mg, 89%). ^1^H NMR (500 MHz, CDCl_3_): δ 8.67 (s, 1H, H2b), 8.30 (s, 1H, H2a), 7.97 (m, 2H, H^Bz^), 7.85 (s, 1H, H8a), 7.78 (s, 1H, H8b), 7.77 (s, 1H, H^Triazole^), 7.46–7.43 (m, 1H, H^Bz^), 7.36–7.32 (m, 2H, H^Bz^), 5.85 (d, *J* = 4.5 Hz, 1H, H1′a), 5.78 (d, *J* = 5.4 Hz, 1H, H1′b), 5.44 (d, *J* = 14.6 Hz, 1H, CH_2_N), 5.36 (d, *J* = 14.6 Hz, 1H, CH_2_N), 5.12 (m, 1H, H2′b), 4.87 (dd, *J* = 5.2, 14.3 Hz, 1H, H5′b), 4.70 (dd, *J* = 7.3, 14.2 Hz, 1H, H5′b), 4.48–4.47 (m, 1H, H3′b), 4.46–4.44 (m, 1H, H2′a), 4.37–4.34 (m, 1H, H4′b), 4.24–4.23 (m, 1H, H3′a), 4.07–4.05 (m, 1H, H4′a), 3.87 (dd, *J* = 4.0, 11.3 Hz, 1H, H5′a), 3.71 (dd, *J* = 3.3, 11.3 Hz, 1H, H5a’), 1.57 (s, 9H, *t*Bu^Boc^), 0.91 (s, 9H, *t*Bu^TBS^), 0.89 (s, 9H, *t*Bu^TBS^), 0.87 (s, 9H, *t*Bu^TBS^), 0.80 (s, 9H, *t*Bu^TBS^), 0.77 (s, 9H, *t*Bu^TBS^), 0.08 (s, 3H, Me^TBS^), 0.07 (s, 6H, Me^TBS^), 0.05 (s, 3H, Me^TBS^), 0.04 (s, 3H, Me^TBS^), 0.02 (s, 3H, Me^TBS^), −0.04 (s, 3H, Me^TBS^), −0.08 (s, 3H, Me^TBS^), −0.18 (s, 3H, Me^TBS^), −0.39 (s, 3H, Me^TBS^). ^13^C NMR (126 MHz, CDCl_3_): δ 176.8 (C=O), 152.9 (C2b), 150.3 (2C, Cq), 149.6 (C=O), 146.7 (2C, C2a and Cq), 145.4 (Cq), 142.6 (C8b), 141.9 (Cq^Triazole^), 139.0 (C8a), 135.6 (Cq), 131.9 (C^Bz^), 129.7 (2C, C^Bz^), 128.1 (2C, C^Bz^), 125.6 (CH^Triazole^), 122.8 (Cq), 122.6 (Cq), 90.4 (C1′b), 88.5 (C1′a), 85.1 (C4′a), 83.6 (C4′b), 82.5 (Cq^Boc^), 76.0 (C2′a), 73.5 (C3′b), 73.3 (C2′b), 71.7 (C3′a), 62.6 (C5′a), 51.9 (C5′b), 43.8 (CH_2_N), 28.2 (3C, *t*Bu^Boc^), 26.1 (3C, *t*Bu^TBS^), 26.0 (3C, *t*Bu^TBS^), 25.9 (3C, *t*Bu^TBS^), 25.8 (3C, *t*Bu^TBS^), 25.7 (3C, *t*Bu^TBS^), 18.6 (Cq^TBS^), 18.2 (Cq^TBS^), 18.1 (Cq^TBS^), 18.0 (Cq^TBS^), 17.9 (Cq^TBS^), −4.2 (Me^TBS^), −4.3 (Me^TBS^), −4.4 (Me^TBS^), −4.5 (Me^TBS^), −4.6 (Me^TBS^), −4.7 (2C, Me^TBS^), −5.0 (Me^TBS^), −5.2 (Me^TBS^), −5.3 (Me^TBS^). HRMS (ESI) *m*/*z*: calcd for C_65_H_108_N_13_O_10_Si_5_ [M − H]^−^: 1370.7188; found: 1370.7138.

Compound **11b**: Following the general procedure A for CuAAC, starting from alkyne **3b** (50 mg, 0.067 mmol) and azido compound **7** (49 mg, 0.08 mmol) and using Cyclohexane/EtOAc 6:4 as eluent for flash chromatography purification, compound **11b** was obtained as a white foam (54 mg, 60%). ^1^H NMR (500 MHz, CDCl_3_): δ 8.72 (s, 1H, H2a or H2b), 8.10 (s, 1H, H2a or H2 b), 8.10 (s, 1H, H8a or H8b), 8.01 (s, 1H, NH), 7.93 (s, 1H, H8a or H8b), 7.90 (s, 1H, H^Triazole^), 5.85–5.83 (m, 2H, H1′a and H1′b), 5.30–5.21 (m, 2H, CH_2_N), 5.17 (dd, *J* = 5.8, 4.3 Hz, 1H, H2′b), 4.92 (dd, *J* = 14.3, 6.5 Hz, 1H, H5′b), 4.62 (dd, *J* = 14.3, 6.5 Hz, 1H, H5′b), 4.48–4.45 (m, 1H, H3′b), 4.39–4.35 (m, 1H, H4′b), 4.32–4.27 (m, 2H, H2′a, H3′a), 4.09–4.06 (m, 1H, H4′a), 3.98 (dd, *J* = 11.6, 3.4 Hz, 1H, H5′a), 3.75 (dd, *J* = 11.6, 2.5 Hz, 1H, H5′a), 1.56 (s, 18H, *t*Bu^Boc^), 0.92 (s, 9H, *t*Bu^TBS^), 0.90 (s, 9H, *t*Bu^TBS^), 0.86 (s, 9H, *t*Bu^TBS^), 0.85 (s, 9H, *t*Bu^TBS^), 0.76 (s, 9H, *t*Bu^TBS^), 0.10 (s, 3H, Me^TBS^), 0.10 (s, 3H, Me^TBS^), 0.07 (s, 3H, Me^TBS^), 0.05 (s, 3H, Me^TBS^), 0.03 (s, 3H, Me^TBS^), 0.01 (s, 3H, Me^TBS^), −0.03 (s, 3H, Me^TBS^), −0.08 (s, 6H, Me^TBS^), −0.40 (s, 3H, Me^TBS^). ^13^C NMR (126 MHz, CDCl_3_): δ 153.0 (C2a or C2b), 150.5 (Cq), 150.4 (Cq), 149.6 (2C, C=O), 146.9 (Cq^Triazole^), 146.5 (C2a or C2b), 144.2 (Cq), 142.7 (Cq), 142.3 (C8a or C8b), 138.1 (C8a or C8b), 125.7 (CH^Triazole^), 123.0 (Cq), 122.3 (Cq), 90.4 (C1′a or C1′b), 88.9 (C1′a or C1′b), 84.1 (C4′a), 83.9 (C4′b), 82.4 (2C, Cq^Boc^), 76.7 (C2′a), 73.5 (C3′b), 73.2 (C2′b), 70.5 (C3′a), 61.7 (C5′a), 51.9 (C5′b), 43.1 (CH_2_N), 28.3 (3C, *t*Bu^Boc^), 28.3 (3C, *t*Bu^Boc^), 26.3 (3C, *t*Bu^TBS^), 26.0 (3C, *t*Bu^TBS^), 25.9 (6C, *t*Bu^TBS^), 25.8 (3C, *t*Bu^TBS^), 18.7 (Cq^TBS^), 18.2 (Cq^TBS^), 18.1 (Cq^TBS^), 18.0 (Cq^TBS^), 17.9 (Cq^TBS^), −4.1 (Me^TBS^), −4.4 (2C, 2 Me^TBS^), −4.5 (Me^TBS^), −4.6 (Me^TBS^), −4.7 (Me^TBS^), −4.9 (Me^TBS^), −5.1 (Me^TBS^), −5.2 (Me^TBS^), −5.3 (Me^TBS^). HRMS (ESI) *m*/*z*: calcd for C_63_H_114_N_13_O_11_Si_5_ [M + H]^+^: 1368.7601; found 1368.7611.

Synthesis of compound **12** following pathway 1: Protected compound **9a** (768 mg, 0.56 mmol) was dissolved in DCM/MeOH 4:1 (10 mL) and MeNH_2_ 33% in EtOH (3.6 mL, 28.0 mmol) was added at 0 °C to the solution. The reaction was stirred at room temperature for 16 h, then diluted in DCM and washed with brine. The combined organic layers were dried over MgSO_4_ and concentrated. The residue was purified by silica gel chromatography (eluent: Cyclohexane/EtOAc 5:5) to provide the debenzoylated compound as a white foam. The residue was then dissolved in DCM (1.4 mL) and ZnBr_2_ (686 mg, 2.80 mmol) was added. The reaction mixture was vigorously stirred at room temperature for 24 h. Then, water (5.8 mL) was added and the reaction mixture was stirred for 2 additional hours. A work up was performed with DCM and brine. The combined organic layers were dried over MgSO_4_ and concentrated. The residue was purified by silica gel chromatography (eluent: Cyclohexane/EtOAc 3:7 then DCM/MeOH 9:1) to afford a yellow powder. The resulting compound was engaged in the last deprotection step and was dissolved in MeOH (10 mL) and CsF (8.5 g, 112 mmol) was added. The reaction mixture was stirred at 60 °C for 24 h, concentrated and diluted in water. The residue was purified by HPLC to afford compound **12** as a white foam (17 mg, 5% over 3 steps). 

Synthesis of compound **12** following pathway 2: Protected compound **9b** (60 mg, 0.046 mmol) was dissolved in DCM (1 mL) and ZnBr_2_ (58 mg, 0.21 mmol) was added. The reaction mixture was vigorously stirred at room temperature for 24 h. Then, water (2 mL) was added and the reaction mixture was stirred for 2 additional hours. A work up was performed with DCM and brine. The combined organic layers were dried over MgSO_4_ and concentrated. The residue was purified by silica gel chromatography (eluent Cyclohexane/EtOAc 2:8) to afford a yellow pale powder. The resulting compound was engaged in the last deprotection step and was dissolved in MeOH (10 mL) before adding CsF (480 mg, 3.2 mmol). The reaction mixture was stirred at 60 °C for 24 h, concentrated and purified by HPLC to afford compound **12** as a white foam (1.2 mg, 4% over 2 steps). 

Compound **12**: ^1^H NMR (500 MHz, (CD_3_)_2_SO): δ 8.35 (s, 1H, H2 or H8), 8.26 (bs, 1 H, NH), 8.23 (s, 1H, H2 or H8), 8.19 (s, 1H, H2 or H8), 8.14 (s, 1H, H2 or H8), 7.81 (s, 1H, H^Triazole^), 7.28 (bs, 2H, NH_2_), 5.90–5.89 (m, 2H, H1′a and H1′b), 5.55 (d, *J* = 5.9 Hz, 1H, OH2′b), 5.43–5.41 (m, 2H, OH2′a and OH3′b), 5.36–5.34 (m, 1H, OH5′a), 5.16 (d, *J* = 4.6 Hz, 1H, OH3′a), 4.74–4.67 (m, 4H, H5′b and CH_2_N), 4.67–4.63 (m, 1H, H2′b), 4.63–4.60 (m, 1H, H2′a), 4.25–4.20 (m, 2H, H4′b and H3′b), 4.16–4.14 (m, 1H, H3′a), 3.97–3.95 (m, 1H, H4′a), 3.69–3.65 (m, 1H, H5′a), 3.58–3.53 (m, 1H, H5′a). ^13^C NMR (126 MHz, (CD_3_)_2_SO): δ 156.1 (Cq), 154.2 (Cq), 152.6 (C2 or C8), 152.2 (C2 or C8), 149.3 (Cq), 148.5 (Cq), 145.3 (Cq), 139.9 (C2 or C8), 139.8 (C2 or C8), 123.6 (CH^Triazole^), 119.8 (Cq), 119.2 (Cq), 87.9 (C1′a or C1′b), 87.7 (C1′a or C1′b), 85.8 (C4′a), 82.5 (C4′b), 73.4 (C2′a), 72.5 (C2′b), 71.0 (C3′b), 70.6 (C3′a), 61.6 (C5′a), 51.3 (C5′b), 35.3 (CH_2_N). HRMS (ESI) *m*/*z*: calcd for C_23_H_26_N_13_O_7_ [M − H]^−^: 596.2078; found: 596.2065. HPLC purity: 96.3%; t_R_ = 17.8 min (MeCN/H_2_O 0:100 to 100:0 over 30 min).

Compound **13**: Protected compound **11a** (400 mg, 0.3 mmol) was dissolved in DCM/MeOH 4:1 (5 mL) and MeNH_2_ 33% in EtOH (1.8 mL, 15.0 mmol) was added at 0 °C to the solution. The reaction was stirred at room temperature for 16 h, then diluted in DCM and washed with brine. The combined organic layers were dried over MgSO_4_ and concentrated. The residue was purified by silica gel chromatography (eluent: EtOAc/MeOH 9:1) to provide the debenzoylated compound as a white foam. The residue was then dissolved in DCM (1 mL) and ZnBr_2_ (117 mg, 0.47 mmol) was added. The reaction mixture was vigorously stirred at room temperature for 24 h. Then, water (2 mL) was added and the reaction mixture was stirred for 2 additional hours. A work up was performed with DCM and brine. The combined organic layers were dried over MgSO_4_ and concentrated. The resulting compound was engaged in the last deprotection step and was dissolved in MeOH (10 mL) and CsF (2.9 g, 19 mmol) was added. The reaction mixture was stirred at 60 °C for 24 h, concentrated and diluted in water. The residue was purified by HPLC to afford compound **13** as a white foam (15 mg, 8% over 3 steps). ^1^H NMR (500 MHz, CD_3_OD): δ 8.35 (2s, 1H, H2a), 8.23 (s, 1H, H8a or H8b), 8.13 (2s, 1H, H8a or H8b), 8.03 (2s, 1H, H2b), 7.84 (s, 0.5H, H^Triazole^), 7.69 (s, 0.5H, H^Triazole^), 6.01–5.98 (m, 1H, H1′a), 5.95–5.93 (m, 1H, H1′b), 5.32 (2s, 2H, CH_2_N), H5′b masked in the residual pick of water, 4.79–4.75 (m, 0.5H, H2′b), 4.71–4.68 (m, 0.5H, H2′b), 4.61 (t, *J* = 5 Hz, 1H, H2′a), 4.47–4.41 (m, 0.5H, H3′b), 4.37–4.31 (m, 1.5H, H3′b and H3′a), 4.14–4.13 (m, 2H, H4′a and H4′b), 3.88–3.85 (m, 1H, H5′a), 3.78–3.74 (m, 1H, H5′a), 3.56 (2s, 3H, CH_3_). ^13^C NMR (126 MHz, CD_3_OD): δ 178.3 (Cq), 163.0 (Cq), 157.3 (Cq), 153.7 (C8a or C8b), 150.4 (Cq), 150.0 (Cq), 149.8 (Cq), 149.4 (Cq), 147.8 (Cq), 147.5 (Cq), 142.0 (C2a or C2b), 126.6 (CH^Triazole^), 125.7 (CH^Triazole^), 126.2 (Cq), 120.8 (Cq), 90.9 (C1′b), 90.4 (C1′a), 87.6 (2C, C4′a and C4′b), 83.9 (C3′a), 76.1 (C2′a), 74.2 (C2′b), 72.2 (C3′b), 62.9 (C5′a), 52.5 (C5′b), 45.1 (CH_2_N), 43.9 (CH_2_N), 37.9 (CH_3_), 35.0 (CH_3_). LRMS (ESI) *m*/*z*: calcd for C_24_H_30_N_13_O_7_ [M + H]^+^: 612.23; found: 612.33. HPLC purity: 95.3%; rt = 14.6 min (MeCN/H_2_O 0:100 to 100:0 over 30 min).

Compound **14**: Compound **11a** (107 mg, 0.08 mmol) was dissolved in DCM (1 mL) and ZnBr_2_ (95 mg, 0.40 mmol) was added. The reaction mixture was vigorously stirred at room temperature for 24 h. Water (2 mL) was added and the reaction mixture was stirred for 2 additional hours. A work up was performed with DCM and brine. The combined organic layers were dried over MgSO_4_ and concentrated. The residue was purified by silica gel chromatography (eluent: Cyclohexane/EtOAc 2:8) to afford a white foam (52 mg). The purified intermediate was then dissolved in MeOH (5 mL) and CsF (1.2 g, 8 mmol) was added. The reaction mixture was stirred at 60 °C for 24 h, concentrated and diluted in water. The residue was purified by HPLC to afford compound **14** as a white foam (7 mg, 13% over 2 steps). ^1^H NMR (500 MHz, CD_3_OD): δ 8.53 (s, 1H, H2 or H8), 8.12 (s, 1H, H2 or H8), 8.06 (s, 1H, H2 or H8), 7.90 (s, 2H, H2 or H8 and H^Triazole^), 7.83–7.81 (m, 2H, H^Bz^), 7.45–7.42 (m, 1H, H^Bz^), 7.33–7.30 (m, 2H, H^Bz^), 5.96 (d, *J* = 5.0 Hz, 1H, H1′a), 5.90 (d, *J* = 5.0 Hz, 1H, H1′b), 5.43 (s, 2H, CH_2_N), H5′b masked in the residual pick of water, 4.64–4.60 (m, 2H, H2′b and H2′a), 4.47 (t, *J* = 5.0 Hz, 1H, H3′b), 4.37–4.34 (m, 1H, H4′b), 4.32–4.30 (m, 1H, H3′a), 4.11 (q, *J* = 3.3 Hz, 1H, H4′a), 3.83 (dd, *J* = 5.0, 15.0 Hz, 1H, H5′a), 3.74 (dd, *J* = 5.0, 15.0 Hz, 1H, H5′a). ^13^C NMR (126 MHz, CD_3_OD): δ 178.8 (C=O), 157.2 (C2 or C8), 153.7 (C2 or C8), 149.2 (Cq), 148.5 (Cq), 146.9 (Cq), 143.3 (Cq), 141.5 (C2 or C8), 141.2 (Cq), 136.7 (C2 or C8), 133.2 (Cq), 130.6 (C^Bz^), 129.1 (2C, C^Bz^), 129.0 (2C, C^Bz^), 127.0 (CH^Triazole^), 123.2 (Cq), 120.6 (Cq), 91.0 (C1′a), 90.4 (C1′b), 87.4 (C4′a), 83.4 (C4′b), 76.0 (C2′a), 74.5 (C2′b), 72.2 (C3′b), 72.0 (C3′a), 62.8 (C5′a), 52.4 (C5′b), 45.0 (CH_2_N). HRMS (ESI) *m*/*z*: calcd for C_30_H_32_N_13_O_8_ [M + H]^+^: 702.2497; found: 702.2470. HPLC purity: 97.1%; t_R_ = 19.0 min (MeCN/H_2_O 0:100 to 100:0 over 30 min).

Compound **15**: Protected compound **11b** (50 mg, 0.036 mmol) was dissolved in DCM (1 mL) and ZnBr_2_ (44 mg, 0.18 mmol) was added. The reaction mixture was vigorously stirred at room temperature for 24 h then water (2 mL) was added and the reaction mixture was stirred for 2 additional hours. A work up was performed with DCM and brine. The combined organic layers were dried over MgSO_4_ and concentrated. The residue was purified by silica gel chromatography (eluent Cyclohexane/EtOAc 1:9) to afford a yellow pale powder. The resulting compound was engaged in the last deprotection step and was dissolved in MeOH (10 mL) and CsF (636 mg, 4.2 mmol) was added. The reaction mixture was stirred at 60 °C for 24 h, concentrated and purified by HPLC to afford compound **15** as a white foam (4.3 mg, 20% over 2 steps). ^1^H NMR (500 MHz, (CD_3_)_2_SO): δ 8.27 (s, 1H, H8b), 8.22 (s, 1H, H2a), 8.15 (s, 1H, H2b), 8.14 (s, 1H, H8a), 7.96 (s, 1H, H^Triazole^), 7.28 (bs, 2H, NH_2_), 5.89 (d, *J* = 5.5 Hz, 1H, H1′b), 5.75 (d, *J* = 5.9 Hz, 1H, H1′a), 5.28–5.17 (m, 2H, CH_2_N), 4.74–4.70 (m, 2H, H5′b), 4.64–4.61 (m, 1H, H2′b), 4.48–4.44 (m, 1H, H2′a), 4.27–4.20 (m, 2H, H3′b and H4′b), 4.10 (d, *J* = 4.9 Hz, 1H, H3′a), 3.92 (q, *J* = 3.8 Hz, 1H, H4′a), 3.64 (dd, *J* = 12.0, 4.0 Hz, 1H, H5′a), 3.53 (dd, *J* = 12.0, 3.9 Hz, 1H, H5′a). ^13^C NMR (126 MHz, (CD_3_)_2_SO): δ 156.1 (Cq), 153.2 (Cq), 152.6 (C2b), 149.3 (Cq), 148.2 (C2a), 142.5 (Cq^Triazole^), 141.2 (Cq), 139.8 (C8b), 137.9 (C8a), 124.5 (CH^Triazole^), 122.8 (Cq), 119.2 (Cq), 87.6 (C1′a or C1′b), 87.6 (C1′a or C1′b), 85.6 (C4′a), 82.3 (C4′b), 73.9 (C2′a), 72.5 (C2′b), 70.9 (C3′b), 70.4 (C3′a), 61.4 (C5′a), 51.4 (C5′a), 41.2 (CH_2_N). HRMS (ESI) *m*/*z*: calcd for C_23_H_28_N_13_O_7_ [M + H]^+^: 598.2229; found: 598.2229. HPLC purity: 96.2%; t_R_ = 17.5 min (MeCN/H_2_O 0:100 to 100:0 over 30 min).

Compound **16**: Propargyl amine (1.4 μL, 0.031 mmol), BOP (13 mg, 0.031 mmol) and DIPEA (6.8 μL, 0.039 mmol) were added at 0 °C to a solution of inosine (7 mg, 0.026 mmol) in DMF (1 mL). The reaction mixture was stirred at room temperature for 24 h and then concentrated under reduced pressure. The crude was diluted in water and purified by HPLC to afford compound **16** as a white foam (5.7 mg, 71%). ^1^H NMR (500 MHz, D_2_O): δ 8.31 (s, 1H, H8), 8.27 (s, 1H, H2), 6.07 (d, *J* = 5 Hz, 1H, H1′), H2′ masked in the residual pick of water, 4.47–4.45 (m, 1H, H3′), 4.35 (s, 2H, CH_2_N), 4.33 (q, *J* = 3.3 Hz, 1H, H4′), 3.97 (dd, *J* = 5, 11 Hz, 1H, H5′), 3.88 (dd, *J* = 5, 10 Hz, 1H, H5′), 2.69 (t, *J* = 2.5 Hz, 1H, C≡CH). ^13^C NMR (126 MHz, D_2_O): δ = 153.9 (C5), 152.3 (C2), 147.8 (C4), 140.4 (C8), 119.6 (C6), 88.4 (C1′), 85.8 (C4′), 80.3 (C≡C-CH_2_), 73.7 (C2′), 71.9 (C≡CH), 70.6 (C3′), 61.5 (C5′), 30.2 (CH_2_N). HRMS (ESI) *m*/*z*: calcd for C_13_H_14_N_5_O_4_ [M − H]^−^: 304.1045; found: 304.1048. 

Compound **17**: Pyridine (610 μL, 7.48 mmol) and thionyl chloride (1.4 mL, 18.7 mmol) were added at 0 °C, over 5 min to a solution of adenosine (1 g, 3.74 mmol) in MeCN (10 mL). The reaction mixture was stirred at 0 °C for 3 h before being warmed to room temperature and stirred for 16 h. The resulting precipitate was filtered and dissolved in water/MeOH (5:1) and aqueous ammonia (25%, 2 mL) was added. The reaction mixture was stirred at room temperature for 30 min and the solvent was removed under reduced pressure to provide 5′-chloroadenosine [66]. The resulting 5′-chloroadenosine was then solubilized in DMF (5 mL) and sodium azide (1.2 g, 18.7 mmol) was added. The reaction mixture was heated at 80 °C for 5 h, and cooled to room temperature. The excess of sodium azide was removed by filtration and the filtrate purified by flash chromatography (DCM/MeOH 9:1) to give **17** as a white foam (393 mg, 36% over 2 steps). ^1^H NMR (500 MHz, CD_3_OD): δ 8.29 (s, 1H, H8), 8.21 (s, 1H, H2), 6.03 (s, 1H, H1′), 4.80–4.78 (m, 1H, H2′), 4.40–4.36 (m, 1H, H4′), 4.27 (q, *J* = 5 Hz, 0.5H, H3′), 4.18 (q, *J* = 5 Hz, 0.5H, H3′), 3.94 (dd, *J* = 10, 5 Hz, 0.5H, H5′), 3.84 (dd, *J* = 10, 5 Hz, 0.5H, H5′), 3.69–3.62 (m, 1H, H5′). HRMS (ESI) *m*/*z*: calcd for C_10_H_13_N_8_O_3_ [M + H]^+^: 293.1110; found: 293.1098. Analytical data were in accordance with the literature [67].

General procedure B for CuAAC reaction: To a solution of alkyne **16** or **18** (1 eq) in DMF (1 mL), were successively added azido compound **17** (1.5 eq), CuSO_4_ (0.3 eq, in water 500 µL) and sodium ascorbate (0.6 eq, in water 500 µL). The mixture was stirred at room temperature for 16 h and then concentrated in vacuo. The crude product was purified by HPLC to afford the desired compounds.

Synthesis of compound **12** following general procedure B for CuAAC: Following the general procedure B, starting from alkyne **16** (5.7 mg, 0.019 mmol) and azido compound **17** (8.2 mg, 0.027 mmol), compound **12** was obtained as a white foam (4 mg, 36%).

Compound **18**: Propargyl bromide (80% in toluene) (143 µL, 1.8 mmol) was added to a solution of adenosine (100 mg, 0.37 mmol) in DMF (1 mL) and the reaction mixture was stirred at 50 °C for 24 h. After removal of the solvent under reduced pressure, the crude was diluted in water and purified by HPLC to afford compound **18** as a white foam (64 mg, 56%). ^1^H NMR (500 MHz, D_2_O): δ 8.73 (s, 1H, H2), 8.61 (s, 1H, H8), 6.20 (d, *J* = 5 Hz, 1H, H1′), 5.27 (s, 2H, CH_2_N), 4.84–4.82 (t, *J* = 5 Hz, 1H, H2′), 4.50 (t, *J* = 5 Hz, 1H, H3′), 4.33–4.31 (m, 1H, H4′), 3.97 (dd, *J* = 5, 10 Hz, 1H, H5′), 3.90 (dd, *J* = 5, 10 Hz, 1H, H5′), 3.19 (bs, 1H, C≡CH). ^13^C NMR (126 MHz, D_2_O): δ 150,2 (C6), 146.9 (C2), 146.8 (C4), 143.4 (C8), 119.6 (C5), 88.6 (C1′), 85.5 (C4′), 78.7 (C≡CH), 74.2 (C2′), 73.1 (C≡CH), 70.1 (C3′), 61.1 (C5′), 40.8 (CH_2_N). HRMS (ESI) *m*/*z*: calcd for C_13_H_16_N_5_O_4_ [M]^+^: 306.1196; found: 306.1193.

Compound **19**: Following the general procedure B for CuAAC, starting from alkyne **18** (6.3 mg, 0.020 mmol) and azido compound **17** (9.1 mg, 0.03 mmol), compound **19** was obtained as a white foam (6.5 mg, 53%). ^1^H NMR (500 MHz, CD_3_OD): δ 8.62 (s, 2H, H2a and H8b), 8.14 (s, 1H, H2b), 8.05 (s, 1H, H8a), 7.96 (s, 1H, H^Triazole^), 6.09 (d, *J* = 5 Hz, 1H, H1′b), 5.95 (d, *J* = 5 Hz, 1H, H1′a), 5.52 (s, 2H, CH_2_N), 4.92–4.96 (m, 1H, H5′b), H5′b masked in the residual pick of water, 4.76–4.74 (m, 1H, H2′a), 4.62 (t, *J* = 5 Hz, 1H, H2′b), 4.48 (t, *J* = 5 Hz, 1H, H3′a), 4.38–4.33 (m, 2H, H3′b and H4′a), 4.15 (q, *J* = 3.1 Hz, 1H, H4′b), 3.89–3.85 (m, 1H, H5′a), 3.76–3.70 (m, 1H, H5′a). ^13^C NMR (126 MHz, CD_3_OD): δ 157.2 (Cq), 154.0 (C2b), 152.2 (Cq), 150.3 (Cq), 148.4 (C2a), 148.0 (Cq), 144.1 (C8b), 141.7 (C8a), 140.8 (Cq^Triazole^), 126.7 (CH^Triazole^), 121.3 (Cq), 120.7 (Cq), 91.2 (C1′a), 90.4 (C1′b), 87.6 (C4′b), 83.6 (C4′a), 76.5 (C2′b), 74.5 (C2′a), 72.4 (C3′a), 71.8 (C3′b), 62.5 (C5′a), 52.7 (C5′b), 45.9 (CH_2_N). HRMS (ESI) *m*/*z*: calcd for C_23_H_28_N_13_O_7_ [M + H]^+^: 598.2234; found: 598.2222. HPLC purity: 97.6%; t_R_ = 14.6 min (MeCN/H_2_O 0:100 to 100:0 over 30 min).

## 4. Conclusions

We reported in this study the synthesis of new SAM-adenosine conjugates with a 1,2,3-triazole linker that covalently links the SAM analogue to the N-6 or N-1 position of the adenosine substrate. The use of protecting groups allowed the formation of the *N*^6^ and 1-*N*-conjugates but required numerous steps of protection and deprotection. Revisiting the synthetic strategy, we were able to avoid the steps of protection and deprotection of the hydroxyl and exocyclic amine functions and to propose more straightforward and efficient syntheses. The *N*^6^ and 1-*N* conjugates were obtained in 2 steps with overall yield of 26% and 30%, respectively. In addition, we developed an efficient methodology based on CuAAC to get access to conjugates by connecting through a triazole linker an analogue of RNA MTases substrate (an adenosine modified at the N-6 or N-1 position) to a SAM analog cofactor. We think that this approach could be applied for the preparation of modified oligonucleotides to get more complex bisubstrate analogues for the study of m^6^A and m^1^A RNA MTases.

## Supplementary Materials

The following are available online: ^1^H and ^13^C NMR spectra of compounds **12**–**15** and **19**, and HPLC chromatograms of compounds **12**–**15** and **19**.
