# Supplementary material for: Synthesis of Triazole-Linked SAM-Adenosine Conjugates: Functionalization of Adenosine at N-1 or N-6 Position without Protecting Groups
Source: Molecules. 2020 Jul 16;25(14):3241. doi: 10.3390/molecules25143241 (PMC7397255; doi:10.3390/molecules25143241)
Supplement: Supplementary file 1 [file molecules-25-03241-s001.pdf]

Supporting Information

Synthesis of triazole-linked SAM-adenosine conjugates: functionalization of adenosine at N1 or N6 position without protecting groups

Colette Atdjian, Dylan Coelho, Laura Iannazzo*, Mélanie Ethève-Quelquejeu*, Emmanuelle Braud*

Laboratoire de Chimie et de Biochimie Pharmacologiques et Toxicologiques, Université de Paris, UMR 8601, Paris, F-75006 France; CNRS UMR 8601, Paris, F-75006, France.

Table of contents

1. Numbering of synthesized molecules S3

2. NMR spectra of final compounds S4

Compound **12** (^{1}H and ^{13}C spectra) S4

Compound **13** (^{1}H and ^{13}C spectra) S6

Compound **14** (^{1}H and ^{13}C spectra) S8

Compound **15** (^{1}H and ^{13}C spectra) S10

Compound **19** (^{1}H and ^{13}C spectra) S12

3. HPLC spectra of final compounds S14

HPLC trace of compound **12** S14

HPLC trace of compound **13** S14

HPLC trace of compound **14** S15

HPLC trace of compound **15** S15

HPLC trace of compound **19** S16

1. Numbering of synthesized molecules

Characterizations of the synthesized compounds are indicated according to the following numbering of atoms:

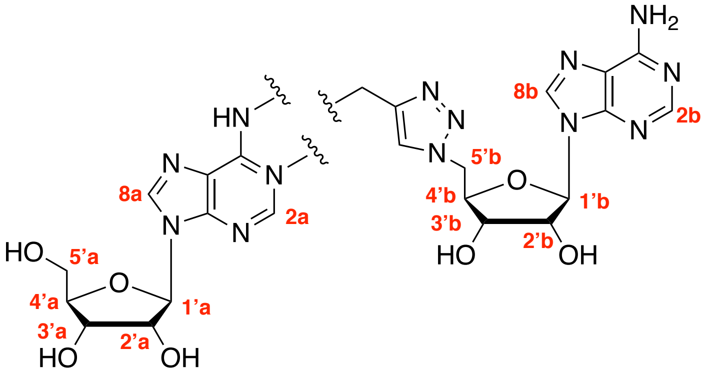

2. NMR spectra of final compounds

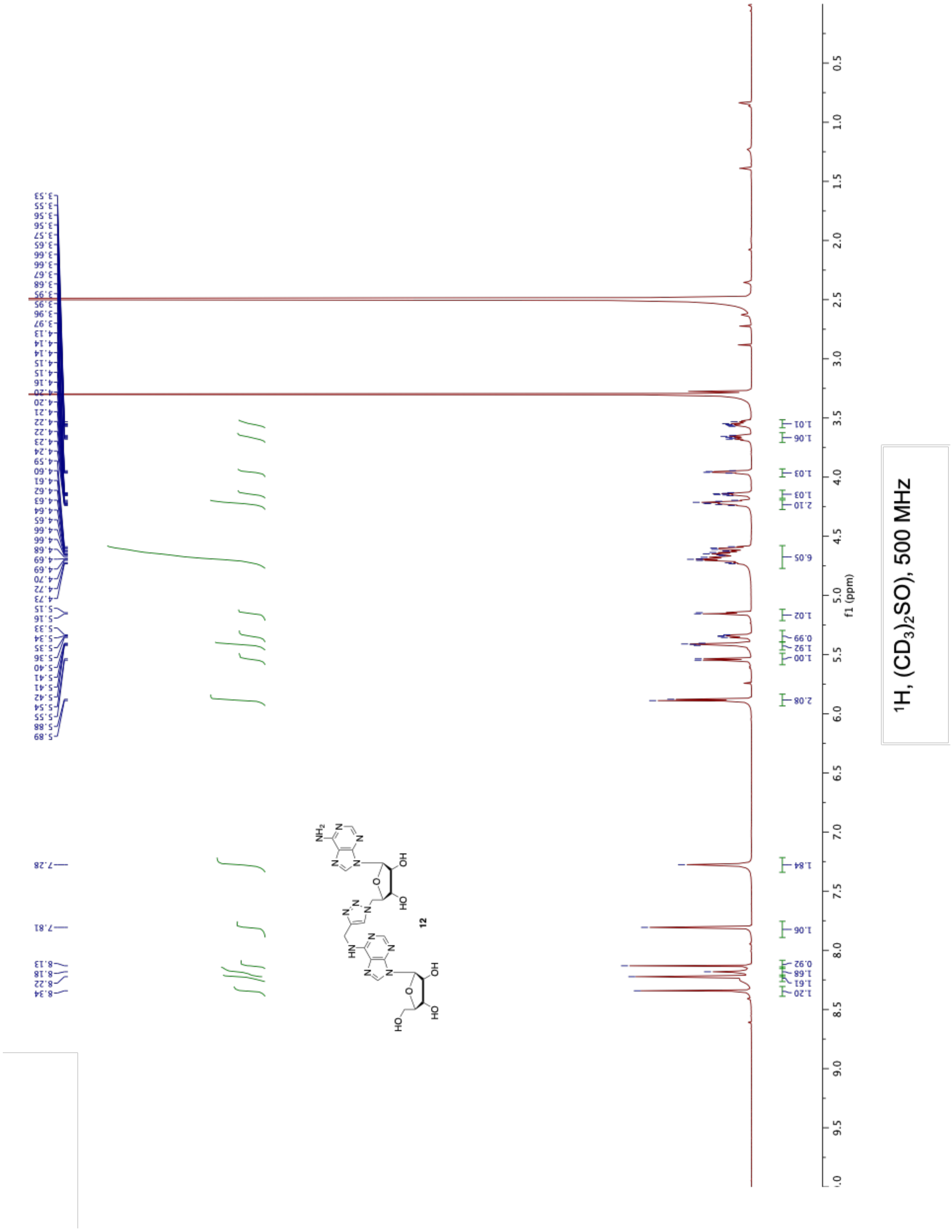

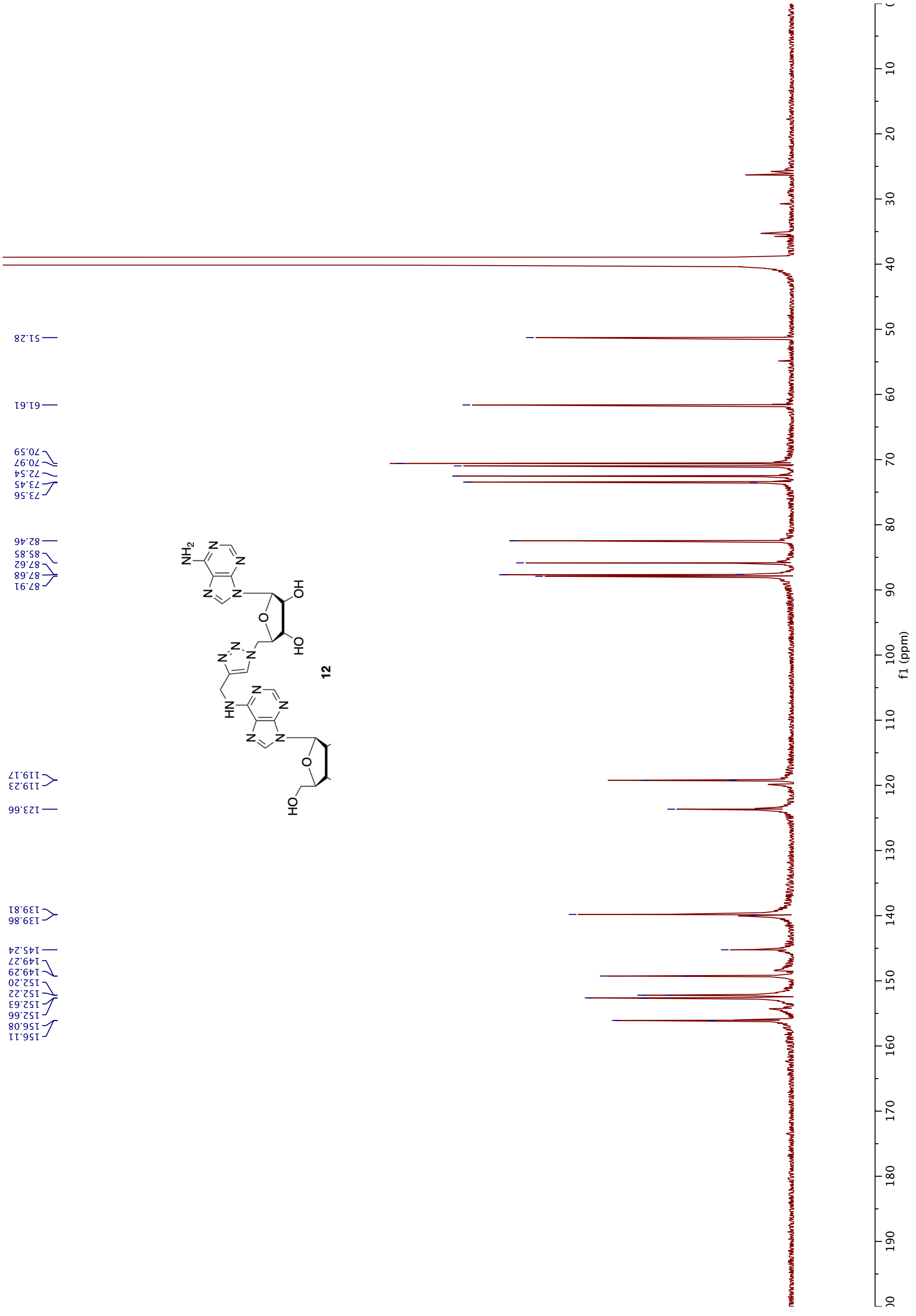

^{13}C, $(CD_3)_2SO$), 126 MHz

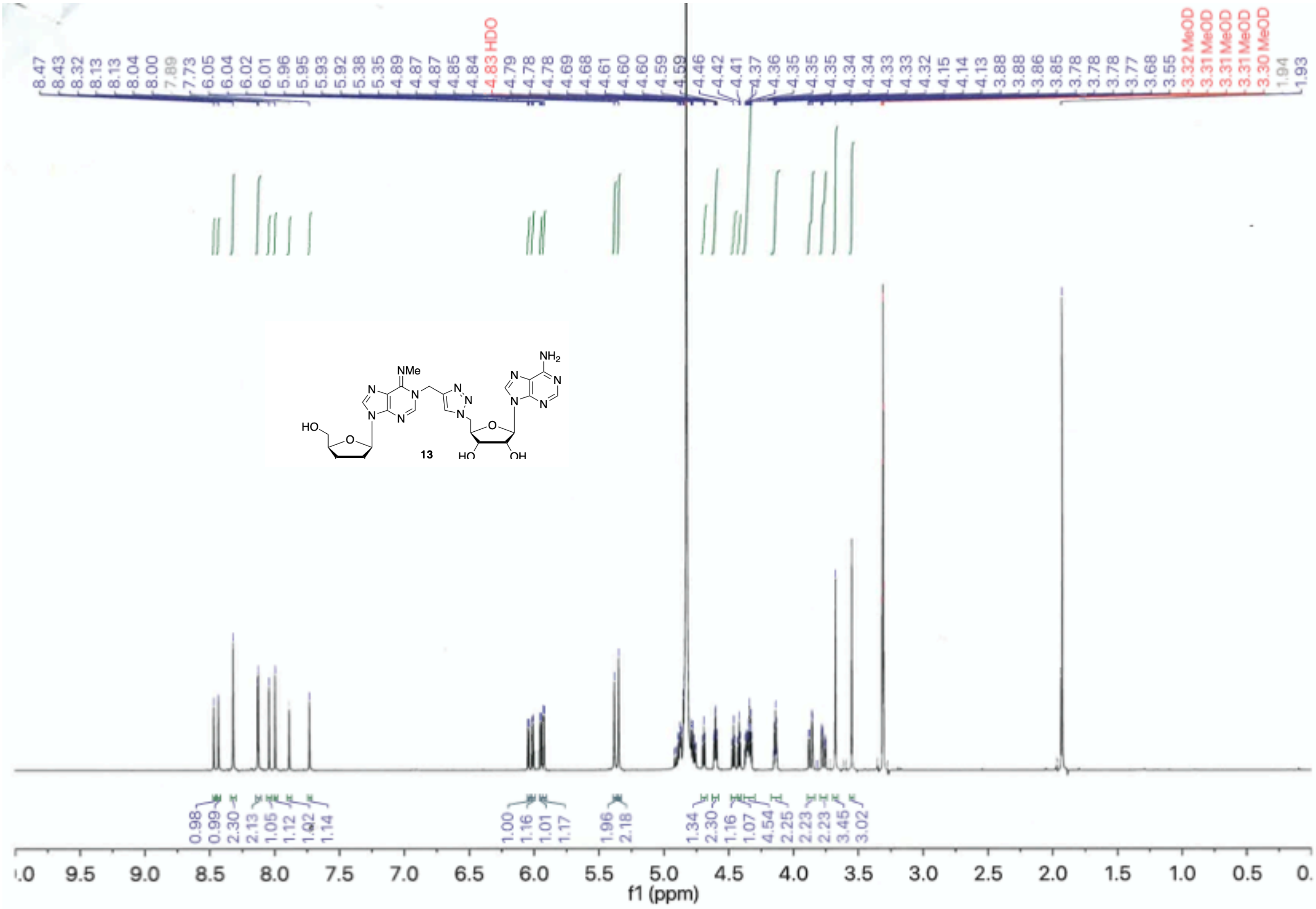

^{1}H, (CD_3OD), 500 MHz

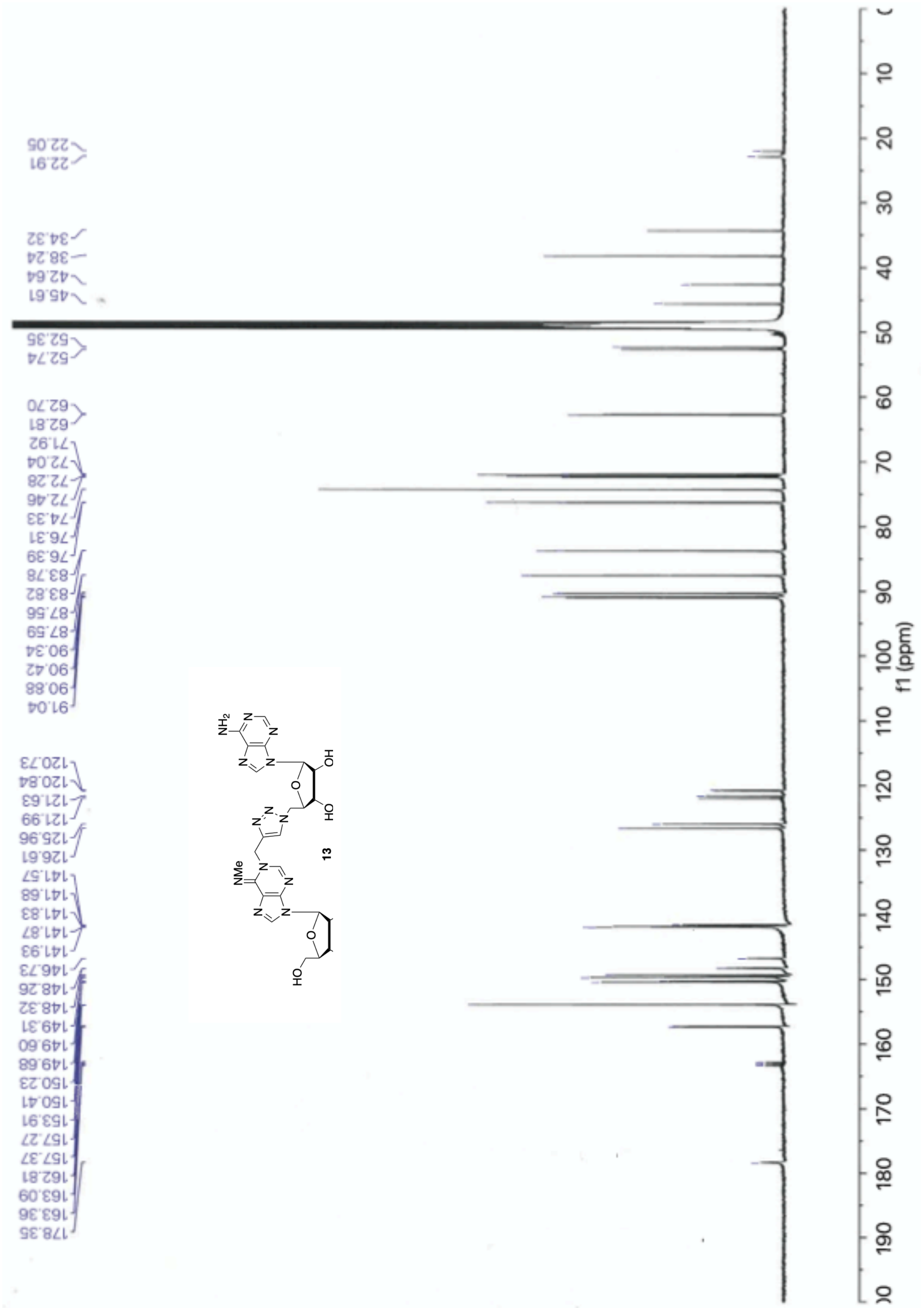

^{13}C, (CD_3OD), 126 MHz

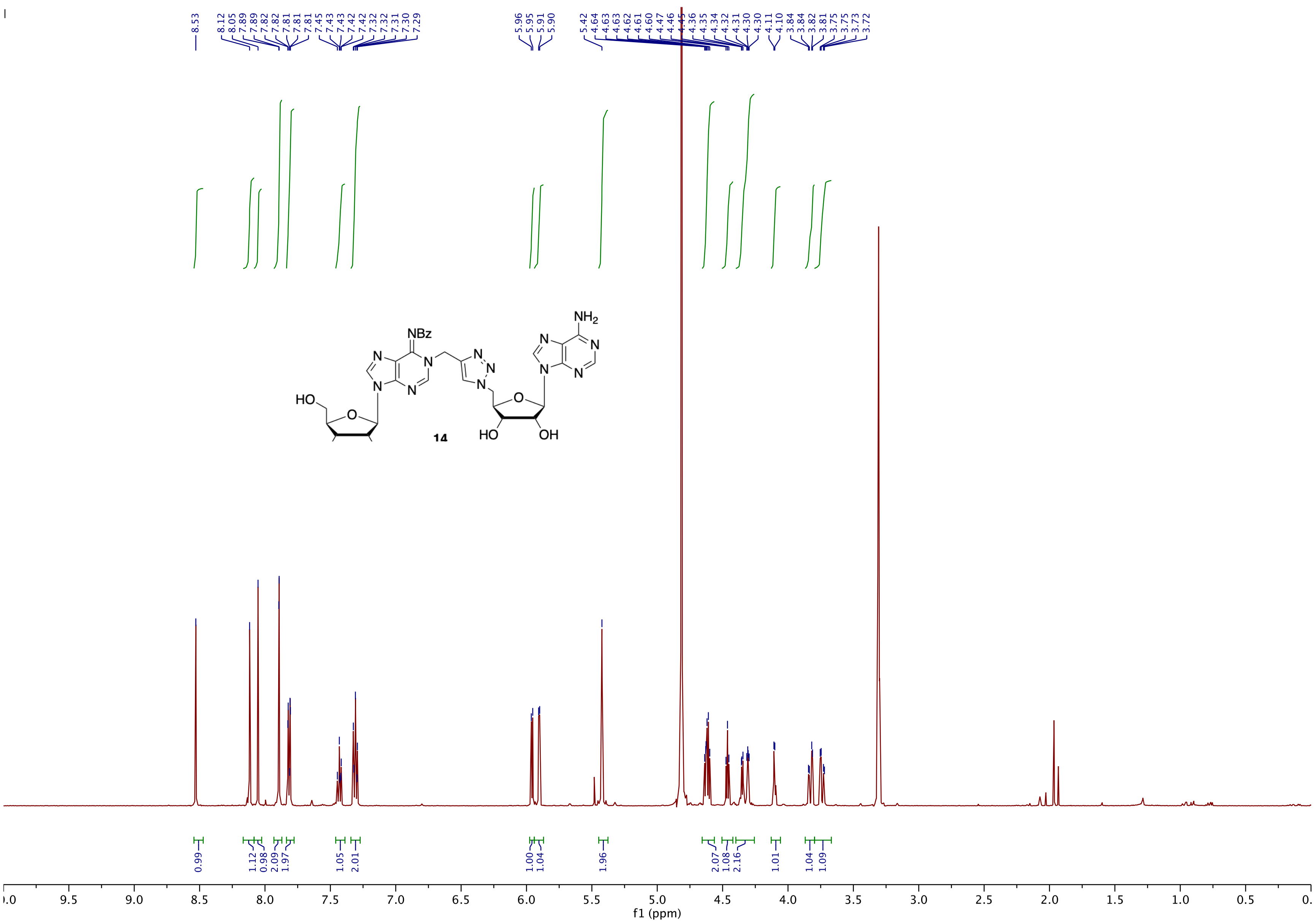

^{1}H, (CD_3OD), 500 MHz

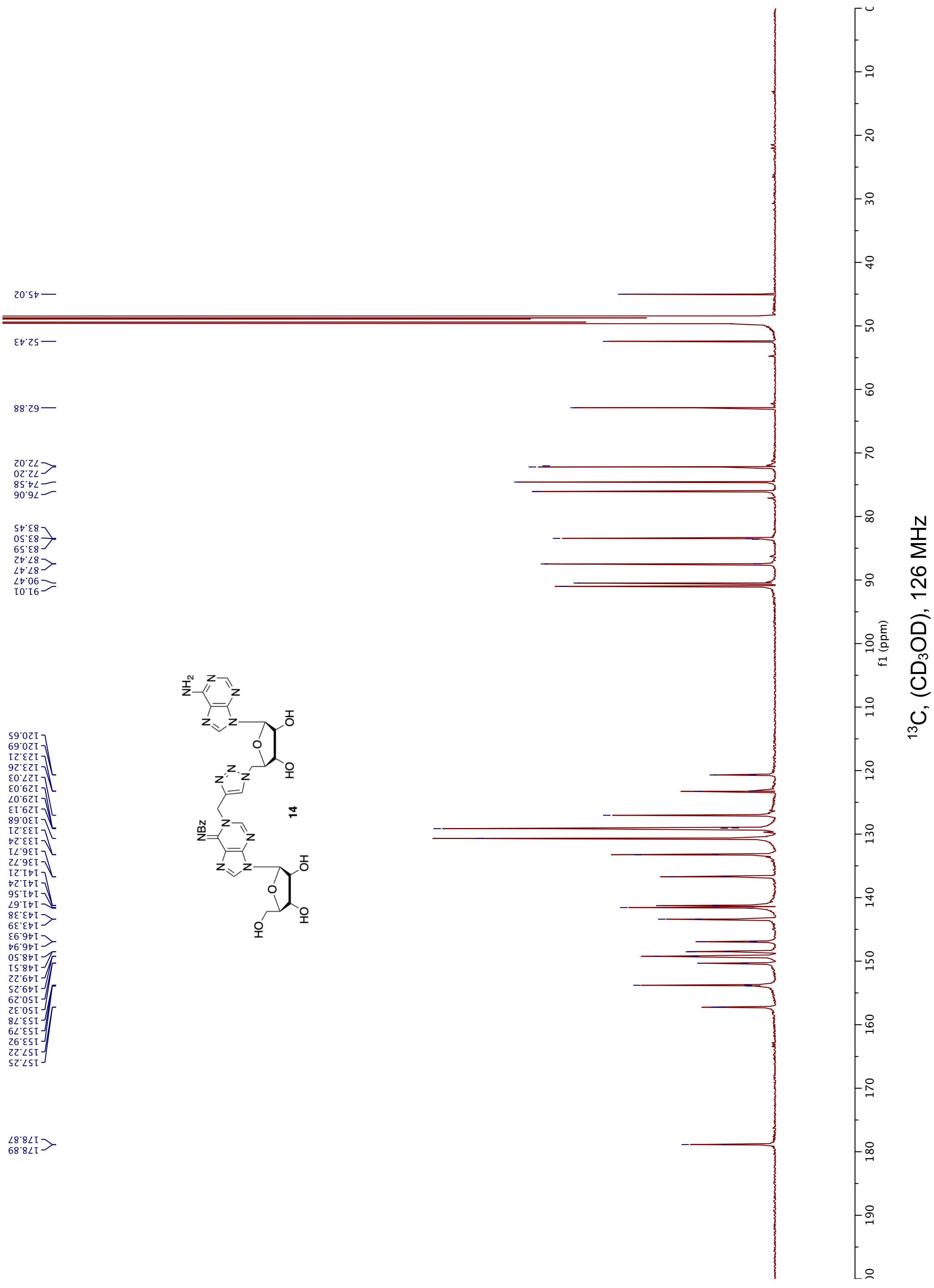

^{13}C, (CD_3OD), 126 MHz
f1 (ppm)
14
NBz
NH_2
45.02
52.43
62.88
72.02
72.20
74.58
76.06
83.45
83.50
83.59
87.42
87.47
90.47
91.01
120.65
120.69
123.21
123.26
127.03
129.03
129.07
129.13
130.68
133.21
133.24
136.71
136.72
141.21
141.24
141.56
141.67
143.38
143.39
146.93
146.94
148.50
148.51
149.22
149.25
150.29
150.32
153.78
153.79
153.92
157.22
157.25
178.87
178.89

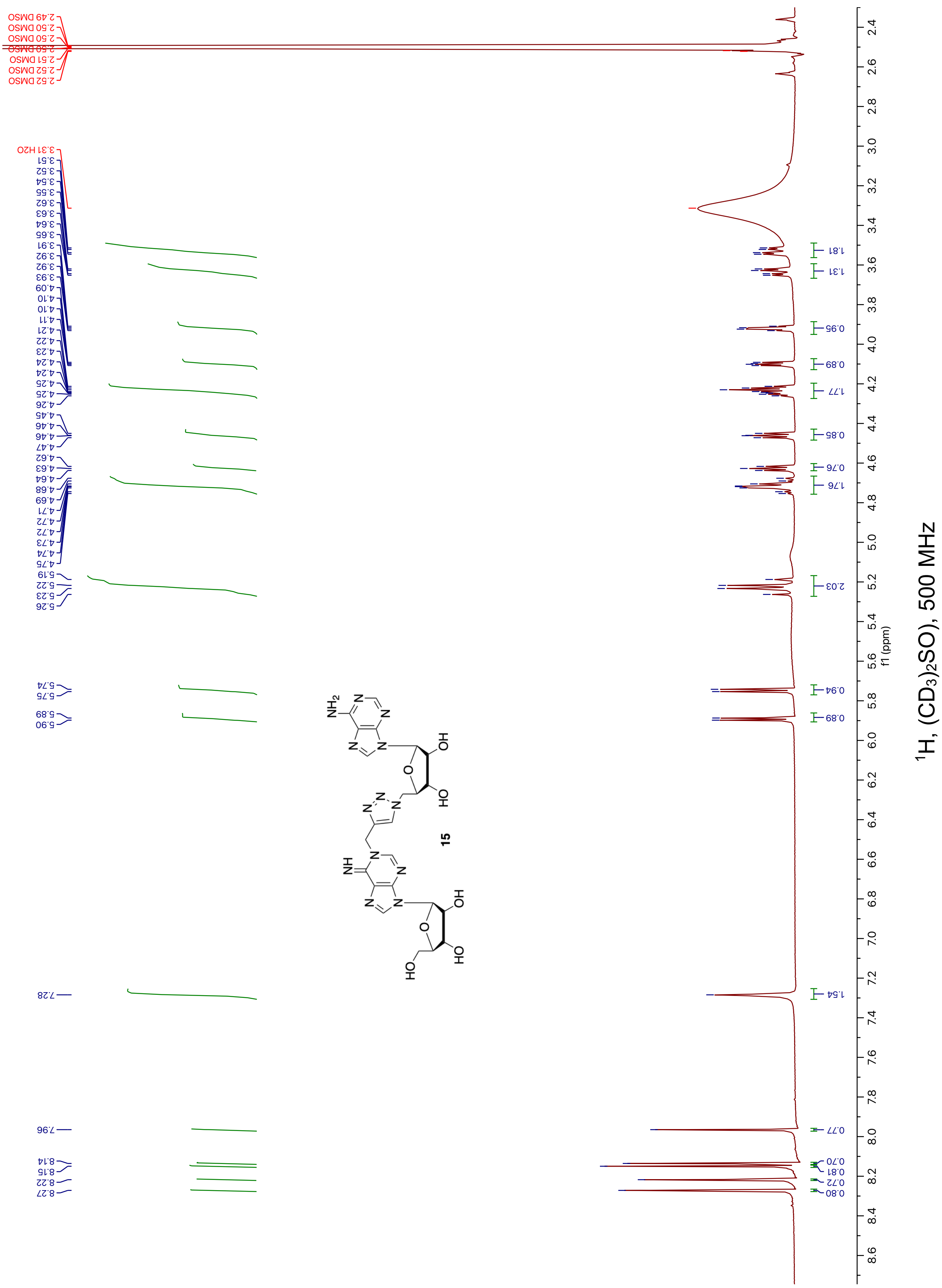

^1H, $(CD_3)_2SO)$, 500 MHz
f1 (ppm)
15

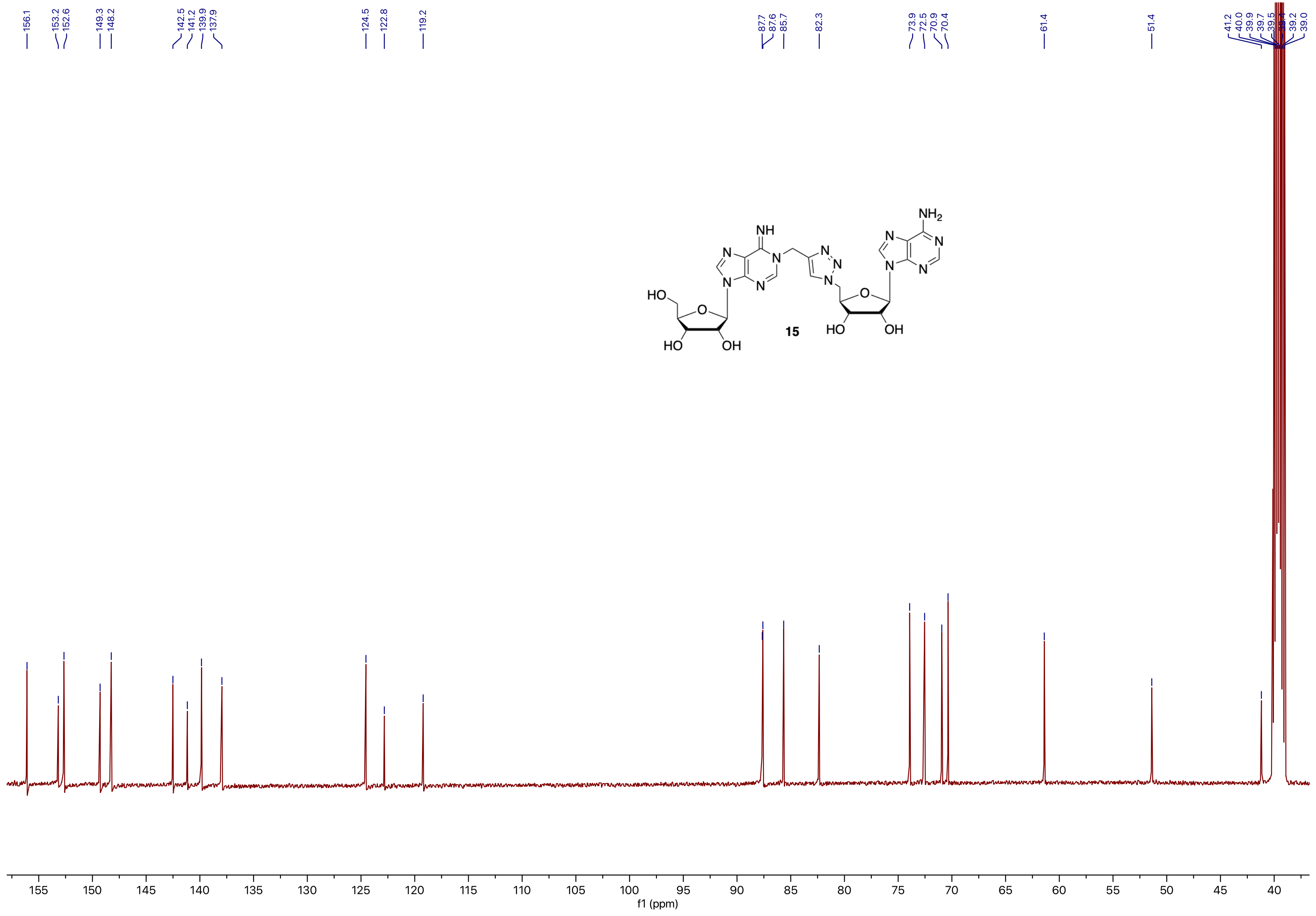

^{13}C, $(CD_3)_2SO)$, 126 MHz

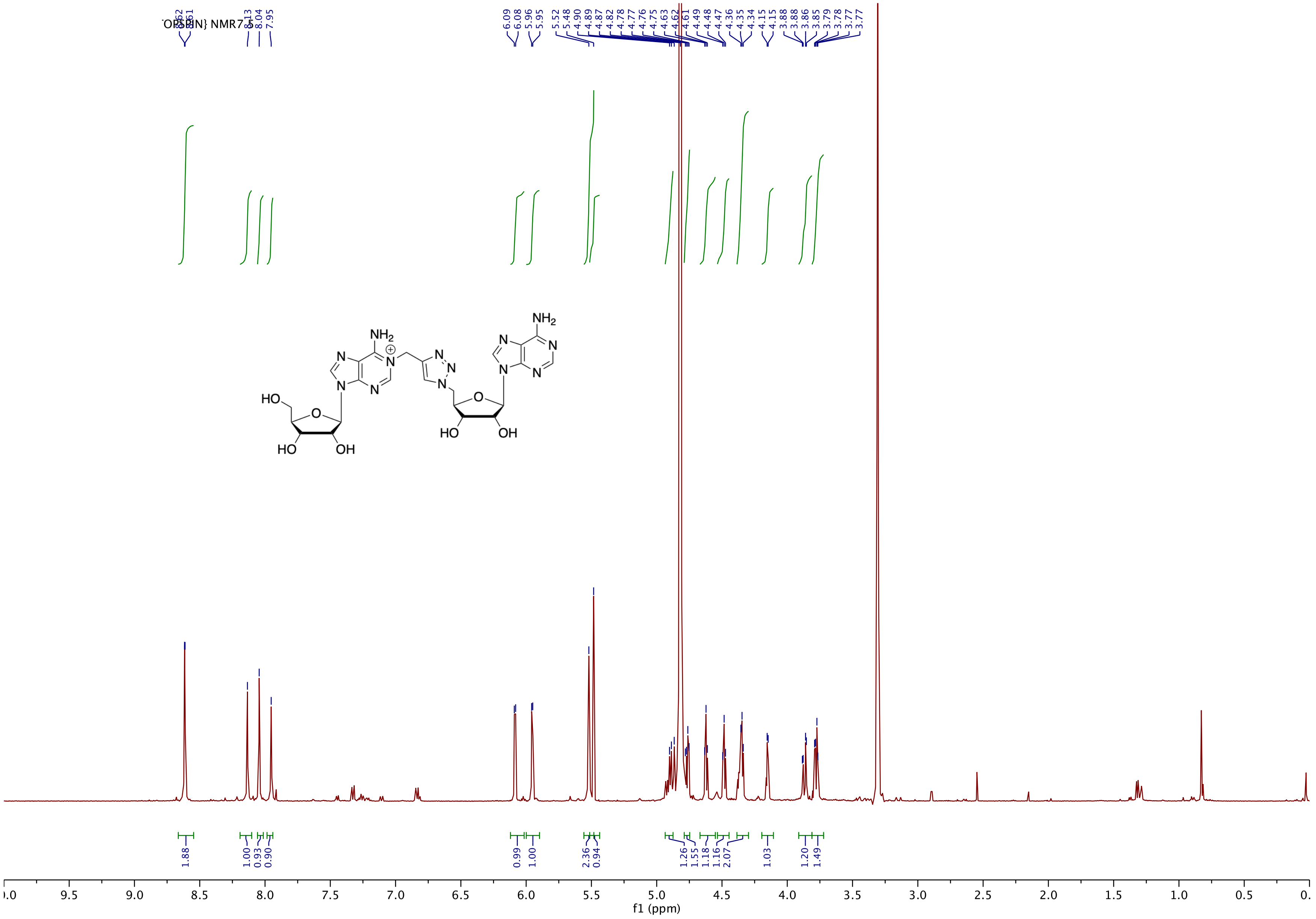

^1H, (CD_3OD), 500 MHz

li1322-cryo.31.1.1r
C13UDEFT1K MeOD {C:\Bruker\TOPSPIN} NMR7.5

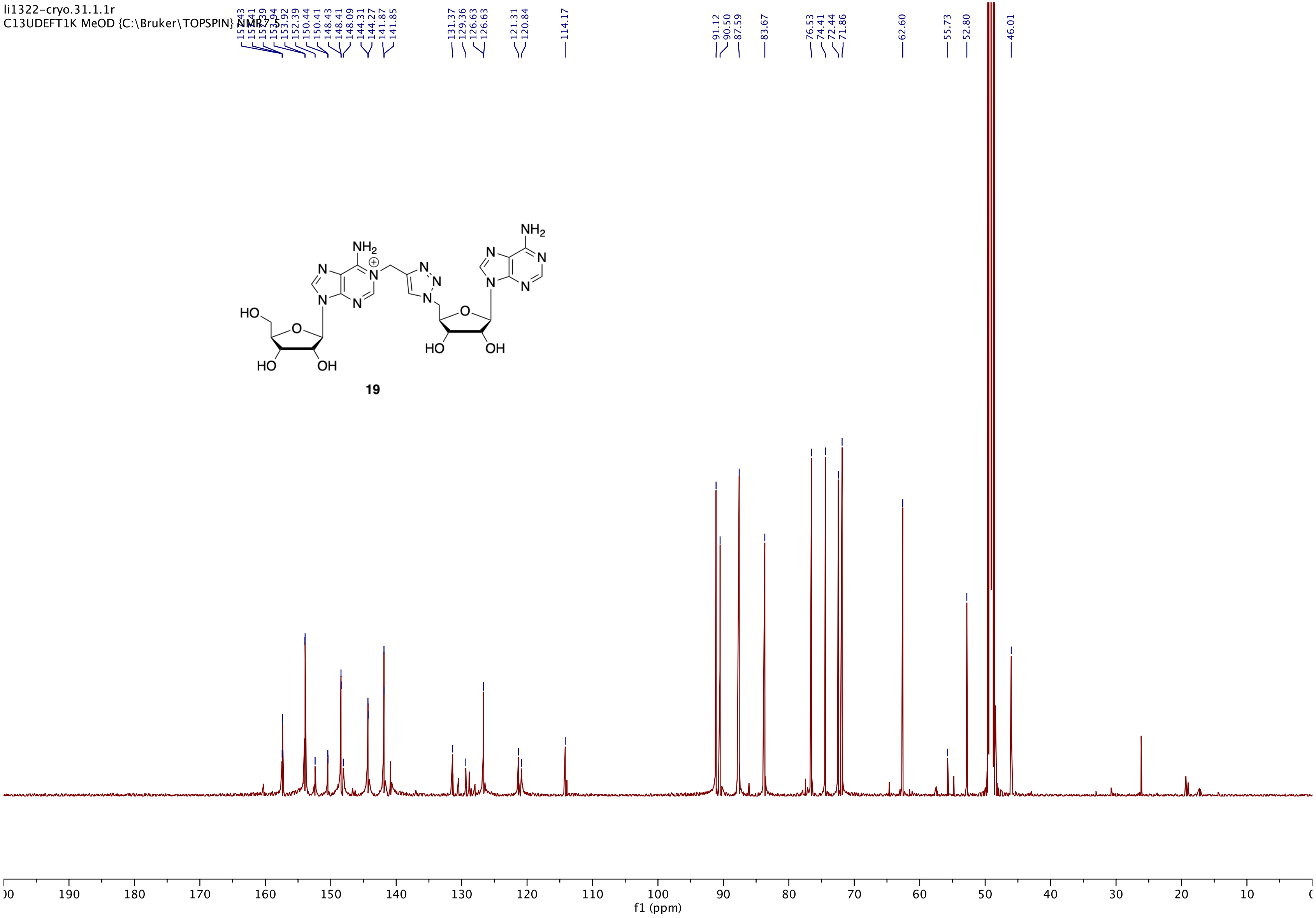

19

^{13}C, (CD_3OD), 126 MHz

3. HPLC spectra of final compounds

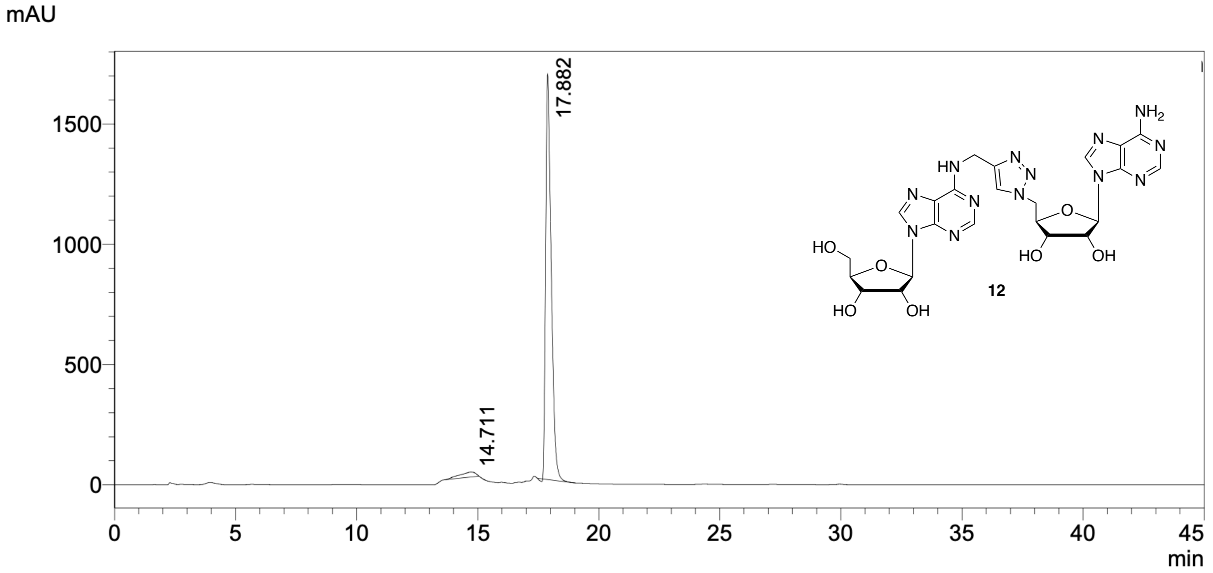

Peak#	Ret. Time	Area	Height	Conc.
1	14.711	1006582	20864	3.628
2	17.882	26738595	1684634	96.372
Total		27745177	1705497	

C18 100-5 NUCLEOSIL (Macherey Nagel), 25 mm X 4.6 mm, 5 μm.
Flow: 1 mL/min; gradient: 0 to 100 % B in 30 min (A: H_2O; B: MeCN).
Detection 254 nm.

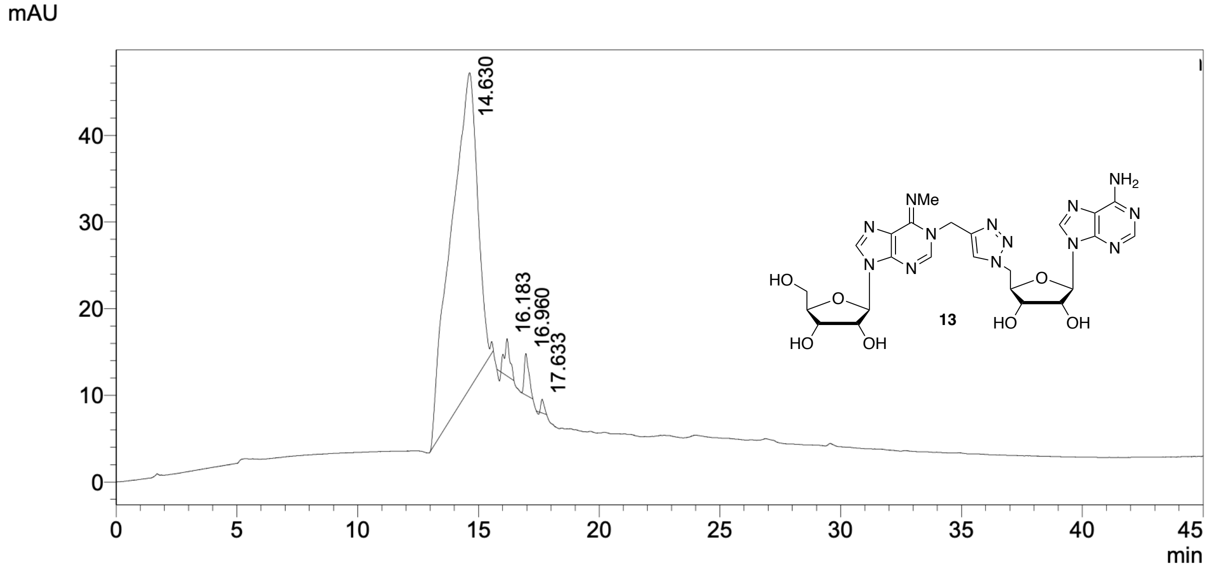

Peak#	Ret. Time	Area	Height	Conc.
1	14.630	2831932	36452	95.346
2	16.183	62509	4320	2.105
3	16.960	62520	4795	2.105
4	17.633	13191	1577	0.444
Total		2970152	47145	

C18 100-5 NUCLEOSIL (Macherey Nagel), 25 mm X 4.6 mm, 5 μm.
Flow: 1 mL/min; gradient: 0 to 100 % B in 30 min (A: H_2O; B: MeCN).
Detection 254 nm.

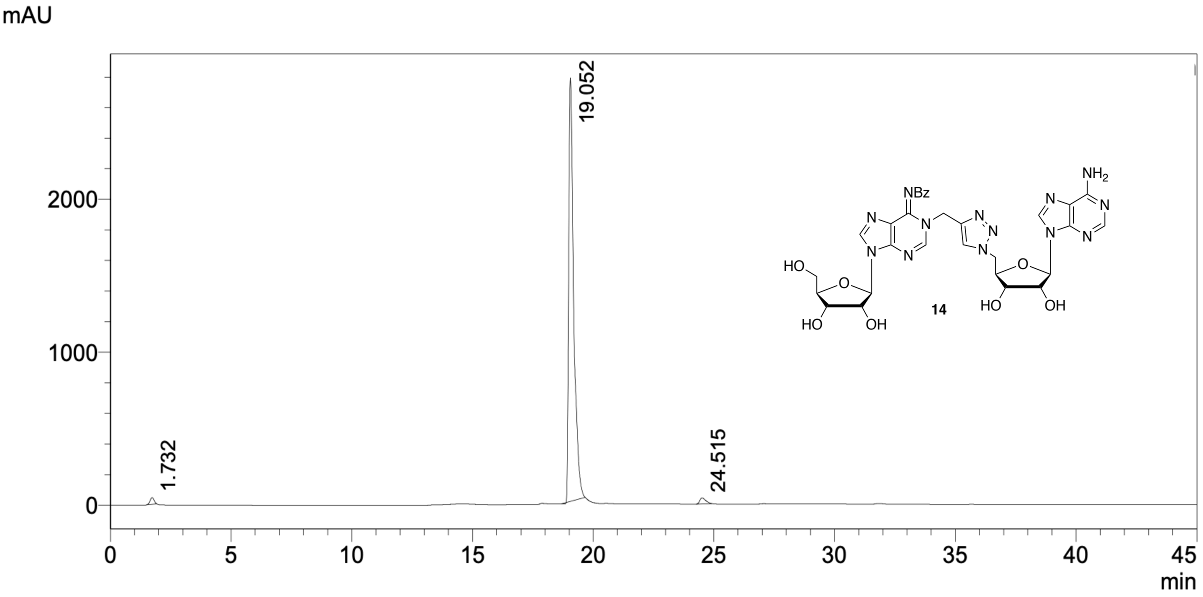

Peak#	Ret. Time	Area	Height	Conc.
1	1.732	506799	43757	1.249
2	19.052	39396210	2769417	97.112
3	24.515	664968	39548	1.639
Total		40567976	2852722	

C18 100-5 NUCLEOSIL (Macherey Nagel), 25 mm X 4.6 mm, 5 μm.

Flow: 1 mL/min; gradient: 0 to 100 % B in 30 min (A: H_2O; B: MeCN).

Detection 214 nm.

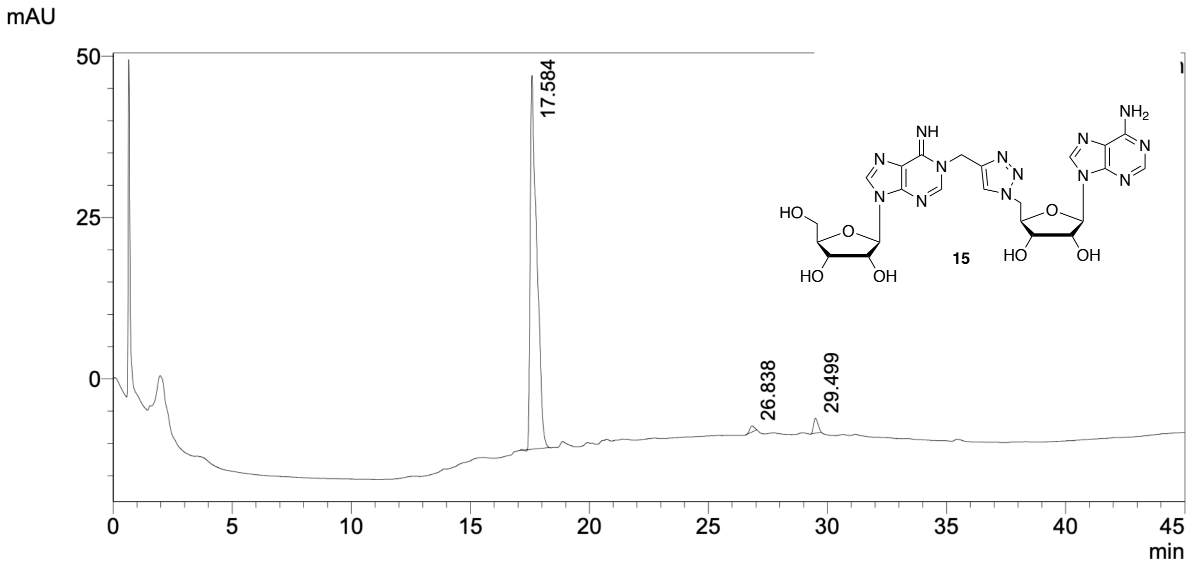

Peak#	Ret. Time	Area	Height	Conc.
1	17.584	1102188	57894	96.275
2	26.838	13201	972	1.153
3	29.499	29450	2288	2.572
Total		1144838	61155	

C18 100-5 NUCLEOSIL (Macherey Nagel), 25 mm X 4.6 mm, 5 μm.

Flow: 1 mL/min; gradient: 0 to 100 % B in 30 min (A: H_2O; B: MeCN).

Detection 214 nm.

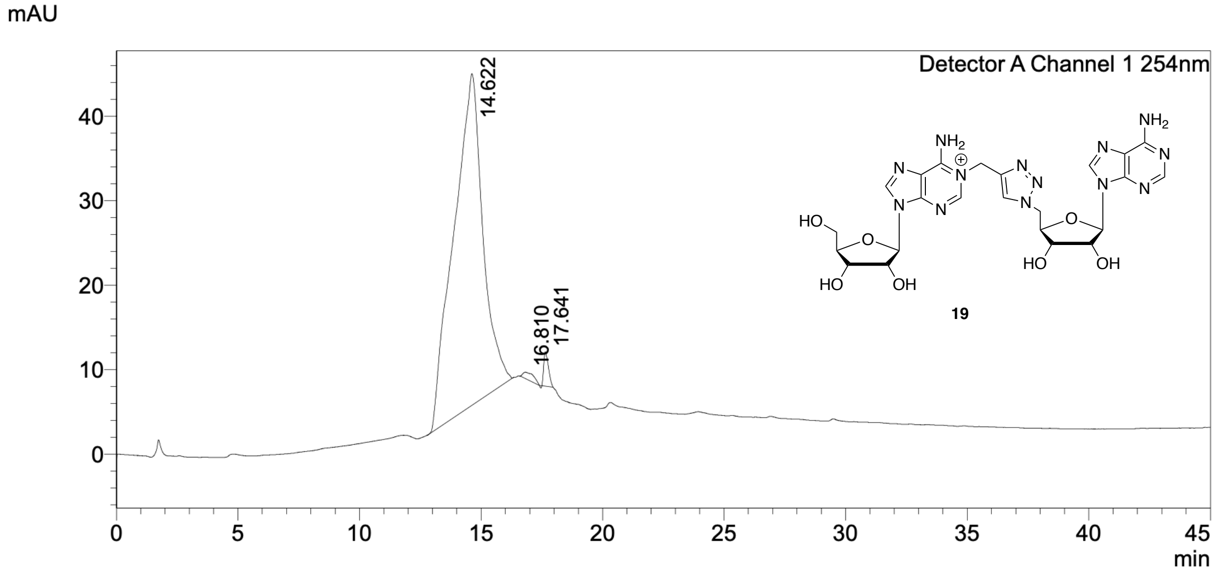

Peak#	Ret. Time	Area	Height	Conc.
1	14.622	3286323	39240	97.614
2	16.810	27588	745	0.819
3	17.641	52749	4035	1.567
Total		3366660	44021	

C18 100-5 NUCLEOSIL (Macherey Nagel), 25 mm X 4.6 mm, 5 μm.

Flow: 1 mL/min; gradient: 0 to 100 % B in 30 min (A: H_2O; B: MeCN).

Detection 254 nm.
